# Awareness and attitudes towards risks of do-it-yourself dental practices among adults attending a dental college in Mangalore city, Karnataka: a cross-sectional study

**DOI:** 10.3389/froh.2026.1945086

**Published:** 2026-10-29

**Authors:** Junaid Junaid, Shaik Saaffaath Muzammil, Rekha P. Shenoy, Imran Pasha Mohammed, Supriya Amanna, Apoorva Kotian, Pramada Prabhakar

**Affiliations:** Department of Public Health Dentistry, Yenepoya Dental College, Yenepoya (Deemed to be University), Mangaluru, Karnataka, India

**Keywords:** do-it-yourself dentistry, health literacy, oral health behaviour, oral health information, patient education, self-care

## Abstract

**Background:**

Increasing availability of dental information, products, and options through digital platforms has contributed to interest in do-it-yourself (DIY) dental practices. This study assessed awareness and attitudes toward DIY dental practices, their prevalence and types, and associations with demographic characteristics.

**Methods:**

A cross-sectional questionnaire-based study was conducted among 294 adults attending the Department of Public Health Dentistry, Yenepoya Dental College, Mangalore, Karnataka, India, between August 2025 and May 2026. Participants were recruited using convenience sampling. Questionnaire assessed demographic characteristics, DIY dental practice use, awareness, and attitudes. Data were analyzed using IBM SPSS Statistics version 27. Non-parametric tests, Spearman's correlation, and multivariable binary logistic regression were performed. Adjusted odds ratios (ORs) with 95% confidence intervals (CIs) were reported.

**Results:**

Of 294 participants, 178 (60.5%) were female and 174 (59.2%) were graduates. Mean awareness and attitude scores were 3.285 ± 0.607 and 3.504 ± 0.718, respectively. Females had significantly higher awareness scores than males (*p* = 0.017), while attitude scores did not differ significantly by gender (*p* = 0.535). Awareness differed significantly across educational groups (*p* = 0.002). DIY dental practice use was reported by 115 participants (39.1%; 95% CI: 33.5%–44.8%). Aligners were the most frequently reported practice (63.5%), followed by home remedies (27.8%), whitening products (11.3%), and oral hygiene products (6.1%). Awareness was positively correlated with attitude (Spearman's *ρ* = 0.624, *p* < 0.001). In multivariable analysis, age (adjusted OR = 1.055, 95% CI: 1.029–1.081; *p* < 0.001) and male gender (adjusted OR = 4.259, 95% CI: 2.442–7.428; *p* < 0.001) were significantly associated with previous DIY use. Educational status was also significantly associated with previous DIY use (*p* = 0.016), whereas awareness was not (adjusted OR = 0.607, 95% CI: 0.329–1.120; *p* = 0.110).

**Conclusion:**

DIY dental practices were commonly reported, with aligner use being the most frequent. Awareness was positively correlated with attitude but was not independently associated with previous DIY use. Given the single-centre convenience sample, findings should not be generalized to the wider population. Further research should examine digital oral health literacy, access to professional care, and informed decision-making regarding consumer-directed dental products.

## 1 Introduction

Oral health is an integral component of overall health and well-being, influencing essential functions such as mastication, speech, aesthetics, and social interaction. Despite advances in preventive and therapeutic dentistry, individuals may seek alternative approaches to manage oral health problems outside conventional dental care. The rapid expansion of internet access and digital health resources has transformed how people obtain health-related information, enabling rapid access to large volumes of oral health content (1). While greater access to information may facilitate public engagement with oral health, online content may also vary in accuracy, completeness, and commercial influence (2, 3). This environment may influence how individuals perceive and make decisions about self-directed oral healthcare, particularly when professional guidance is not sought.

Do-it-yourself (DIY) dental practices refer to self-performed dental procedures or oral health interventions undertaken without direct supervision by a qualified dental professional (4). These practices encompass a wide spectrum of activities, ranging from home-based remedies and over-the-counter tooth-whitening products to more invasive procedures, including orthodontic tooth movement using mail-order aligners, temporary restorations, and impression techniques promoted through online platforms (4, 5). Individuals may consider such practices because of perceived affordability and convenience, limited access to professional dental services, dental anxiety, previous experiences, or exposure to health information outside formal healthcare settings. While certain home-based oral hygiene practices may serve as adjuncts to routine oral care, unsupervised interventions intended to diagnose, prevent, or treat oral diseases raise concerns regarding their safety, effectiveness, and potential for delayed professional care.

The use of DIY dental practices has raised concerns because some procedures are performed without appropriate clinical examination, definitive diagnosis, or professional supervision. Although some commercially available products are intended for home use when used according to appropriate instructions, other self-directed interventions may involve inappropriate materials, techniques, or indications and may expose individuals to preventable complications. Reported concerns include enamel surface damage, dentin hypersensitivity, gingival injury, malocclusion, temporomandibular dysfunction, delayed diagnosis of underlying oral diseases, and irreversible damage resulting from inappropriate materials or techniques (6–8). These risks are particularly relevant when complex dental procedures are simplified through online tutorials, potentially creating the impression that professional dental procedures can be safely replicated at home. Consequently, understanding public perceptions of the risks and acceptability of DIY dental practices is important for evidence-based oral health education and professional guidance.

Digital and social media platforms have become important sources of health-related information, including information about oral health and dental products (9, 10). Online content may include educational material, personal experiences, commercial recommendations, and demonstrations of self-directed dental practices. However, the quality and scientific basis of such information may vary, and individuals may encounter difficulty distinguishing evidence-based information from promotional or misleading content (2, 3). In the present study, perceptions regarding social-media influence were explored through questionnaire items; however, the frequency, duration, platform-specific exposure, and temporal relationship between social-media exposure and DIY dental practice were not directly assessed. Therefore, social media is considered a contextual factor in this study rather than a demonstrated causal determinant of DIY dental behaviour.

Although awareness of the potential risks associated with DIY dentistry has increased in recent years, the available literature has primarily focused on specific practices such as self-administered orthodontics, tooth whitening, self-medication, and the quality of online oral health information (11, 12). Fewer studies have examined public awareness and attitudes toward DIY dental practices as a broader behavioural phenomenon, particularly in low- and middle-income countries, where affordability, accessibility, and digital health literacy may substantially influence oral healthcare decisions. Furthermore, evidence describing how demographic characteristics relate to awareness, attitudes, and the adoption of DIY dental practices remains limited. Generating such evidence is important for developing targeted oral health education strategies, promoting evidence-based decision-making, and supporting public health initiatives to reduce unsafe self-directed dental practices. Therefore, this study aimed to assess awareness and attitudes toward the risks of DIY dental practices among adults attending a dental college in Mangalore City, Karnataka, and to examine their associations with selected demographic characteristics and reported use of DIY dental practices.

## 2 Materials and methods

### 2.1 Study design and setting

This cross-sectional, questionnaire-based study was conducted between August 2025 and May 2026 among adults attending the Department of Public Health Dentistry at Yenepoya Dental College, Mangalore, Karnataka, India. The study protocol was reviewed and approved by the Scientific Review Board of Yenepoya Dental College (Approval Number: 095/PG/SS/074) and the Institutional Ethics Committee (Approval Number: YEC2/2025/200) before commencement of participant recruitment.

### 2.2 Study population and eligibility criteria

The study population comprised adults seeking dental care at the Department of Public Health Dentistry during the study period. Individuals aged 18 years or older who could understand the study questionnaire in English, Kannada, or Malayalam and provided informed consent were eligible to participate. Participants were recruited using convenience sampling from the Department of Public Health Dentistry during the study period. Eligible individuals attending the Department of Public Health Dentistry during the study period were invited to participate. Individuals who agreed to participate provided electronic informed consent and were then provided with a QR code to access the electronic questionnaire. Healthcare professionals from the medical and dental professions were excluded to minimize potential bias arising from their professional knowledge of oral health. Individuals with cognitive impairment or other conditions that could interfere with questionnaire completion were excluded.

### 2.3 Sample size and sampling technique

The sample size was calculated using the single-proportion formula, assuming a 95% confidence level, a 6% margin of error, and an anticipated population proportion of 50%. A 6% margin of error was selected to obtain a feasible sample size within the available study period and recruitment setting while maintaining an acceptable level of precision for this exploratory cross-sectional study. The use of a 50% anticipated proportion was considered a conservative assumption in the absence of a reliable prior estimate for the study population. Based on these assumptions, the minimum required sample size was 267 participants. After accounting for a 10% anticipated non-response rate, the target sample size was increased to 294 participants. The sample size calculation was reviewed and verified by a statistician from the Department of Statistics, Yenepoya (Deemed to be University), before commencement of the study. Participants were recruited using convenience sampling from adults attending the Department of Public Health Dentistry until the target sample size was reached.

### 2.4 Questionnaire development and validation

Data were collected using a structured, self-administered questionnaire developed following a review of the available literature on do-it-yourself (DIY) dental practices, their potential risks, and related oral health behaviours. The questionnaire was initially prepared in English and subsequently translated into Kannada and Malayalam to facilitate participation among individuals with different language preferences. The questionnaire included sociodemographic characteristics, a question assessing previous use of a DIY dental product or method, an open-ended question asking participants who reported previous use to specify the type of DIY practice undertaken, and 5-point Likert-scale statements addressing perceptions and attitudes toward DIY dental practices. The Likert-scale response options ranged from strongly disagree to strongly agree. The awareness items were designed to assess participants' recognition and understanding of the potential risks, consequences, and characteristics of DIY dental practices, whereas the attitude items assessed perceptions, acceptability, preferences, and willingness regarding DIY dental practices and professional dental car The complete questionnaire is provided as Supplementary Material S1.

Content validity was assessed by three subject experts with relevant experience in public health dentistry and oral healthcare. The experts reviewed the questionnaire items for relevance and clarity, and discrepancies were resolved through discussion and consensus before finalization. The questionnaire was administered electronically through Google Forms following electronic informed consent. Because the questionnaire was developed specifically for this study, expert-based content validation was distinguished from broader psychometric validation. The internal consistency of the items included in the awareness and attitude scales was assessed using Cronbach's alpha, and the results are reported in the Statistical Analysis and Results sections. Formal construct validation using exploratory or confirmatory factor analysis and test–retest reliability assessment were not performed.

### 2.5 Scoring of awareness and attitude scales

Item responses were coded from 1 (strongly disagree) to 5 (strongly agree). For analysis, responses were summed within each scale and divided by the number of items to obtain a mean composite score, resulting in a possible score range of 1–5 for both awareness and attitude. Higher scores indicated greater awareness and more favourable attitudes, respectively. Negatively worded items were reverse-scored before computation of the composite scores, where applicable. As no validated cut-off values were established for these study-specific scales, composite scores were analysed as continuous variables and were not categorized as low, moderate, or high. Because the composite scores were calculated as the mean of multiple Likert-scale items within each domain, they were treated as approximately continuous summary measures for descriptive purposes; however, given their non-normal distributions, non-parametric statistical tests were used for inferential analyses.

### 2.6 Data collection

Eligible adult patients attending the Department of Public Health Dentistry were approached consecutively during the study period and were informed about the objectives and procedures of the study. Individuals who agreed to participate accessed the questionnaire by scanning a Quick Response (QR) code linked to a Google Forms survey. The participant information sheet and electronic informed consent form were provided before access to the questionnaire. Participation was voluntary, and eligible individuals who did not wish to participate were not enrolled. Recruitment continued until 294 completed questionnaires were obtained. A total of 294 participants completed the questionnaire and were included in the final analysis. The questionnaire required approximately 15–20 min to complete. No personally identifiable information, including names or contact details, was collected, thereby maintaining participant anonymity.

### 2.7 Outcome measures

The primary outcomes were participant's awareness and attitudes toward the risks of do-it-yourself (DIY) dental practices, assessed using composite scores derived from the relevant 5-point Likert-scale items. The response options ranged from strongly disagree to strongly agree. The secondary outcome was self-reported previous use of a DIY dental product or method, assessed using a dichotomous question (yes/no). Participants who reported previous use were also asked to specify the type of DIY dental practice undertaken through an open-ended question. Sociodemographic characteristics, including age, gender, and educational status, were recorded as explanatory variables.

#### 2.7.1 Scoring methodology

All Likert-scale items were scored on a five-point scale, ranging from 1 (strongly disagree) to 5 (strongly agree). The composite awareness score was calculated as the arithmetic mean of 10 items (Q7 and Q13–Q21), while the composite attitude score was calculated as the arithmetic mean of seven items (Q23–Q27, Q29 and Q30). Both composite scores therefore ranged from 1 to 5, with higher scores representing higher awareness and more favourable attitudes, respectively. Q8–Q12, Q22 and Q28 were analysed separately as individual attitude-related items and were not included in the composite attitude score. Awareness items were intended to assess participants' recognition or understanding of the potential risks, consequences, and characteristics of DIY dental practices, whereas attitude items assessed participants' perceptions, acceptability, preferences, and willingness regarding DIY dental practices and professional dental care.

### 2.8 Statistical analysis

All 294 questionnaires included in the final analysis were complete for the study variables. No item-level missing responses were identified; therefore, no imputation or other missing-data procedures were required. Data were analysed using IBM SPSS V27 Statistics. Categorical variables were summarized using frequencies and percentages, while continuous variables were summarized using mean and standard deviation or median and interquartile range, as appropriate. Item-level responses to the awareness and attitude questions were presented as frequencies and percentages for each response category.

The internal consistency of the awareness and attitude measures was assessed using Cronbach's alpha. The awareness score was derived from the items included in the awareness domain, while the attitude score was derived from the attitude items demonstrating acceptable internal consistency. For the separately analysed attitude/perception items, the following items were reverse-coded for the corresponding reliability analysis: “I feel confident using home remedies for dental problems” (Q8), “I believe DIY dental treatments are convenient” (Q9), “DIY dental options are more affordable than professional dental care” (Q10), “It is acceptable to try DIY solutions when dental clinics are not easily accessible” (Q11), “I am open to using DIY methods if I cannot afford professional dental care” (Q12), “I trust dental products recommended by social media influencers” (Q22), and “DIY treatments are safe if instructions are followed carefully” (Q28). These items were additionally analysed and reported individually using their response frequencies and percentages. No reverse scoring was applied to the final composite attitude scale comprising Q23–Q27, Q29 and Q30.

The distribution of the composite awareness and attitude scores was assessed using the Shapiro–Wilk test and visual inspection of histograms. As both composite scores showed non-normal distributions, non-parametric tests were used for inferential analysis. The Mann–Whitney *U*-test was used to compare awareness and attitude scores between two independent groups, while the Kruskal–Wallis *H*-test was used to compare scores across more than two independent groups. For statistically significant Kruskal–Wallis results, Bonferroni-adjusted pairwise comparisons were performed to identify the groups contributing to the overall difference. For non-parametric group comparisons, effect sizes were reported alongside *p*-values. For Mann–Whitney *U*-tests, rank-biserial correlation (rᵣb) was used as the effect-size measure, while epsilon-squared (*ε*^2^) was used for Kruskal–Wallis tests. Ninety-five percent confidence intervals were reported for the main effect estimates where applicable.

Spearman's rank-order correlation was used to assess the relationship between age and the composite awareness and attitude scores, and to examine the association between awareness and attitude scores. For the open-ended question on the type of DIY dental practice used, responses were categorized into relevant practice groups and summarized descriptively. As multiple responses were permitted, percentages were calculated using the number of participants who reported previous DIY use as the denominator and could therefore exceed 100% when summed. A two-sided *p*-value < 0.05 was considered statistically significant. No missing data were identified for the variables included in the analyses; therefore, no imputation was required.

A multivariable binary logistic regression analysis was performed to examine factors associated with reported previous use of DIY dental practices. Previous DIY use was coded as the binary dependent variable (0 = no, 1 = yes). The independent variables included age, gender, educational status, awareness score, and attitude score. These variables were included based on their potential relevance to reported DIY dental practice use and their availability in the study dataset. The enter method was used to include all specified variables simultaneously. Adjusted odds ratios (AORs) with 95% confidence intervals (CIs) and *p*-values were reported. Multicollinearity was assessed using tolerance and variance inflation factor (VIF) values. Model fit was evaluated using the Hosmer–Lemeshow goodness-of-fit test, and model explanatory power was summarized using Nagelkerke's R^2^.

#### 2.8.1 Use of artificial intelligence tools

Artificial intelligence (AI)-assisted tools, including ChatGPT (OpenAI) and Grammarly AI, were used exclusively to improve the language, grammar, clarity, and organization of the manuscript. These tools were not used for study design, data collection, data analysis, interpretation of results, or formulation of scientific conclusions. All AI-assisted content was critically reviewed, verified, and edited by the authors, who take full responsibility for the accuracy, originality, and integrity of the manuscript.

## 3 Results

### 3.1 Participant characteristics

A total of 294 participants, with no missing responses for the variables were included in the final analysis. Because the study used convenience sampling and the number of eligible individuals approached was not systematically recorded, a formal response rate could not be calculated. Complete data were available for all the participants, and no missing data were present for the variables included in the analyses. The study population comprised 178 females (60.5%) and 116 males (39.5%). With respect to educational status, 174 participants (59.2%) were graduates, 59 (20.1%) had intermediate/pre-university education, 40 (13.6%) were postgraduates, 14 (4.8%) had high-school education, and 7 (2.4%) had no formal education (Table 1).

**Table 1:** Sociodemographic characteristics of the study participants (*N* = 294).

Variable	N	%
Gender		
Male	116	39.5
Female	178	60.5
Educational status		
No formal education	7	2.4
High school	14	4.8
Intermediate/puc	59	20.1
Graduate	174	59.2
Postgraduate	40	13.6

PUC, Pre-University Course; *n*, number of participants; %, percentage.

**Table 3:** Distribution of responses to attitude statements regarding DIY dental practices.

S. nO.	Attitude statement	Strongly disagree *N* (%)	Disagree *N* (%)	Neutral *N* (%)	Agree *N* (%)	Strongly agree *N* (%)
1	It is acceptable to use DIY dental methods when dental clinics are inaccessible	22 (7.5)	37 (12.6)	75 (25.5)	82 (27.9)	78 (26.5)
2	I would use DIY dental methods if I could not afford professional dental care	13 (4.4)	30 (10.2)	75 (25.5)	90 (30.6)	86 (29.3)
3	I prefer professional dental care over DIY dental treatments	22 (7.5)	33 (11.2)	76 (25.9)	135 (45.9)	28 (9.5)
4	I would avoid DIY dental treatments unless advised by a dental professional	17 (5.8)	38 (12.9)	114 (38.8)	82 (27.9)	43 (14.6)
5	Professional dental care is worth the cost for safe and effective treatment	22 (7.5)	42 (14.3)	70 (23.8)	122 (41.5)	38 (12.9)
6	DIY dental treatments should be better regulated	14 (4.8)	40 (13.6)	85 (28.9)	70 (23.8)	85 (28.9)
7	I am likely to discourage others from using unsafe DIY dental techniques.	15 (5.1)	36 (12.2)	82 (27.9)	76 (25.9)	85 (28.9)

DIY, Do-It-Yourself. The composite attitude score was calculated from the seven internally consistent items (Q23–Q27, Q29 and Q30). The seven-item scale had a mean of 3.504 ± 0.718.).

### 3.2 Awareness toward do-it-yourself dental practices

The participants demonstrated varying levels of awareness regarding the risks and potential consequences of do-it-yourself (DIY) dental practices. Item-level responses varied across the awareness statements, with neutral responses common for several items. The 10-item awareness scale demonstrated acceptable internal consistency (Cronbach's *α* = 0.780) (Table 2). The composite awareness score had a mean of 3.285 ± 0.607, median of 3.20 (IQR: 0.60), and range of 1.00–5.00. The Shapiro–Wilk test indicated that the awareness score was not normally distributed (*p* < 0.001); therefore, non-parametric tests were used for subsequent comparisons. The overall distributions of awareness and attitude scores among the study participants are presented in Figure 1.

**Figure 1:**
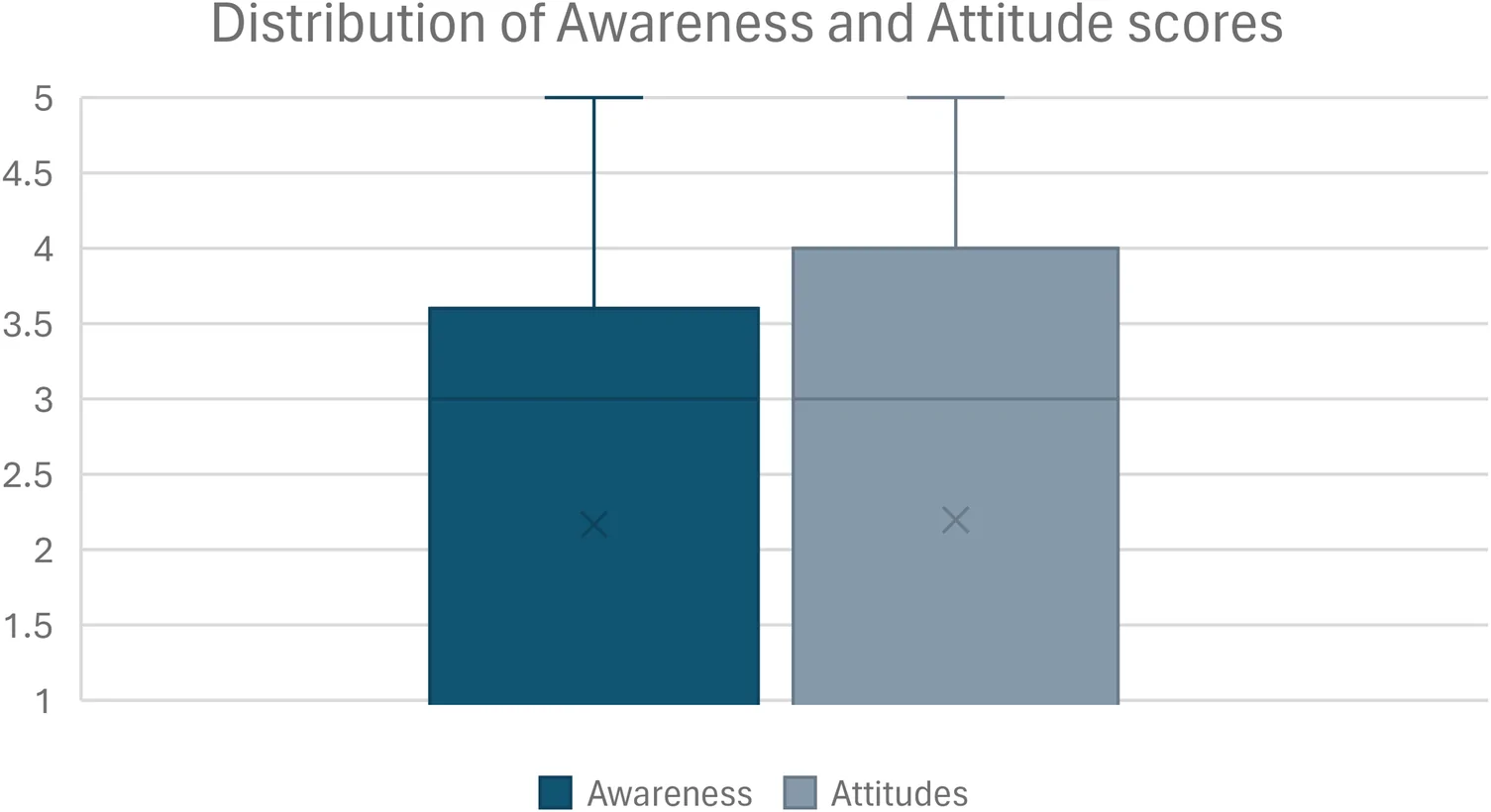
Distribution of awareness and attitude scores among the study participants (*N* = 294).

**Table 2:** Distribution of responses to awareness statements regarding DIY dental practices.

S. No.	Awareness statement	Strongly disagree *N* (%)	Disagree *N* (%)	Neutral *N* (%)	Agree *N* (%)	Strongly agree *N* (%)
1	DIY dental treatments can lead to complications such as tooth damage or gum injury	23 (7.8)	109 (37.1)	74 (25.2)	80 (27.2)	8 (2.7)
2	Most DIY dental methods lack professional guidance	24 (8.2)	41 (13.9)	82 (27.9)	113 (38.4)	34 (11.6)
3	Self-treatment may result in infections and delayed diagnosis	23 (7.8)	31 (10.5)	91 (31.0)	120 (40.8)	29 (9.9)
4	I have heard of individuals experiencing adverse effects following DIY dental treatments	22 (7.5)	42 (14.3)	87 (29.6)	118 (40.1)	25 (8.5)
5	Most people do not understand the risks before attempting DIY dental treatments	16 (5.4)	32 (10.9)	73 (24.8)	140 (47.6)	33 (11.2)
6	DIY dental options are more affordable than professional care	14 (4.8)	39 (13.3)	86 (29.3)	122 (41.5)	33 (11.2)
7	I would prefer professional consultation over self-treatment	21 (7.1)	34 (11.6)	80 (27.2)	120 (40.8)	39 (13.3)
8	I feel confident using home remedies for dental problems	17 (5.8)	75 (25.5)	75 (25.5)	89 (30.3)	38 (12.9)
9	It is acceptable to try DIY dental solutions when dental services are inaccessible	11 (3.7)	40 (13.6)	127 (43.2)	80 (27.2)	36 (12.2)
10	DIY dental treatments should be better regulated	16 (5.4)	38 (12.9)	114 (38.8)	90 (30.6)	36 (12.2)

DIY, Do-It-Yourself. The composite awareness score was calculated from these 10 items (Q7, Q13–Q21); Cronbach's *α* = 0.780).

### 3.3 Attitudes toward do-it-yourself dental practices

Participants generally demonstrated favourable attitudes toward professional dental care and measures aimed at reducing unsafe DIY dental practices. The attitude responses indicated support for professional consultation, regulation of DIY practices, and public awareness initiatives. The seven-item attitude scale demonstrated acceptable internal consistency (Cronbach's *α* = 0.752). The composite attitude score had a mean of 3.504 ± 0.718, median of 3.50 (IQR: 1.00), and range of 1.00–5.00. The Shapiro–Wilk test demonstrated a non-normal distribution (*p* < 0.001). The seven-item attitude scale demonstrated acceptable internal consistency and was used to derive the composite attitude score. In addition to the seven items included in the composite attitude scale, Q8–Q12, Q22 and Q28 were retained as individual attitude items and were analysed descriptively. Neutral responses were common across several of these items. For Q8, 45.6% of participants reported a neutral response, while 43.2% were neutral for Q9 and 40.5% for Q10. Similarly, 39.1% reported a neutral response for Q11. For Q12, the proportions selecting neutral and agree were 27.9% and 28.2%, respectively. For Q22, 33.0% agreed with the statement, whereas 28.9% selected a neutral response for Q28. The complete distribution of responses to these additional individual attitude items is presented in Table 3A. These items were not included in the composite attitude score and were therefore presented separately to avoid conflating their descriptive responses with the composite scale.

**Table 3A:** Distribution of responses to additional individual attitude items regarding DIY dental practices (*N* = 294).

S. nO.	Attitude statement	Strongly disagree *N* (%)	Disagree *N* (%)	Neutral *N* (%)	Agree *N* (%)	Strongly agree *N* (%)
1	I feel confident using home remedies for dental problems.	20 (6.8)	62 (21.1)	134 (45.6)	62 (21.1)	16 (5.4)
2	I believe DIY dental treatments are convenient.	18 (6.1)	50 (17.0)	127 (43.2)	70 (23.8)	29 (9.9)
3	DIY dental options are more affordable than professional dental care.	26 (8.8)	45 (15.3)	119 (40.5)	86 (29.3)	18 (6.1)
4	It is acceptable to try DIY solutions when dental clinics are not easily accessible.	30 (10.2)	49 (16.7)	115 (39.1)	77 (26.2)	23 (7.8)
5	I am open to using DIY methods if I cannot afford professional dental care.	26 (8.8)	78 (26.5)	82 (27.9)	83 (28.2)	25 (8.5)
6	I trust dental products recommended by social media influencers.	33 (11.2)	61 (20.7)	79 (26.9)	97 (33.0)	24 (8.2)
7	DIY treatments are safe if instructions are followed carefully.	22 (7.5)	74 (25.2)	85 (28.9)	81 (27.6)	32 (10.9)

DIY, Do-It-Yourself. These items were analysed as individual attitude items and were not included in the seven-item composite attitude score.).

### 3.4 Prevalence and types of DIY dental practices

Among the 294 participants, 115 (39.1%; 95% CI: 33.5%–44.8%)) reported previous use of DIY dental practices, while 179 (60.9%) reported no previous use. The prevalence of reported DIY dental practice use was therefore 39.1%. Among participants who reported previous DIY use, the most frequently reported practice was use of aligners 73 (63.5%), followed by home remedies 32 (27.8%), whitening kits/products 13 (11.3%), and oral hygiene products 7 (6.1%). Because participants could report more than one type of DIY practice, the percentages are based on the 115 participants who reported previous DIY use and therefore do not sum to 100% (Table 4). The range of reported practices indicates that DIY dental behaviour encompassed both home-based remedies and commercially available dental products, with aligner use accounting for the largest proportion among those reporting previous DIY use.

**Table 4:** Types of DIY dental practices reported among participants with previous DIY use (*n* = 115).

Type of diy dental practice	*N*	%
Aligners	73	63.5
Home remedies	32	27.8
Whitening kits/products	13	11.3
Oral hygiene products	7	6.1

Multiple responses were permitted; therefore, percentages do not sum to 100%. *n*, number of participants; %, frequency.

### 3.5 Comparison of awareness and attitude scores according to gender

Female participants had significantly higher awareness scores than male participants (Mann–Whitney U = 8,625.50, Z = −2.392, rᵣb = 0.165, *p* = 0.017; Figure 2). In contrast, attitude scores did not differ significantly between males and females (Mann–Whitney U = 9,884.50, Z = −0.621, rᵣb = 0.042, *p* = 0.535; Figure 3) (Table 5).

**Figure 2:**
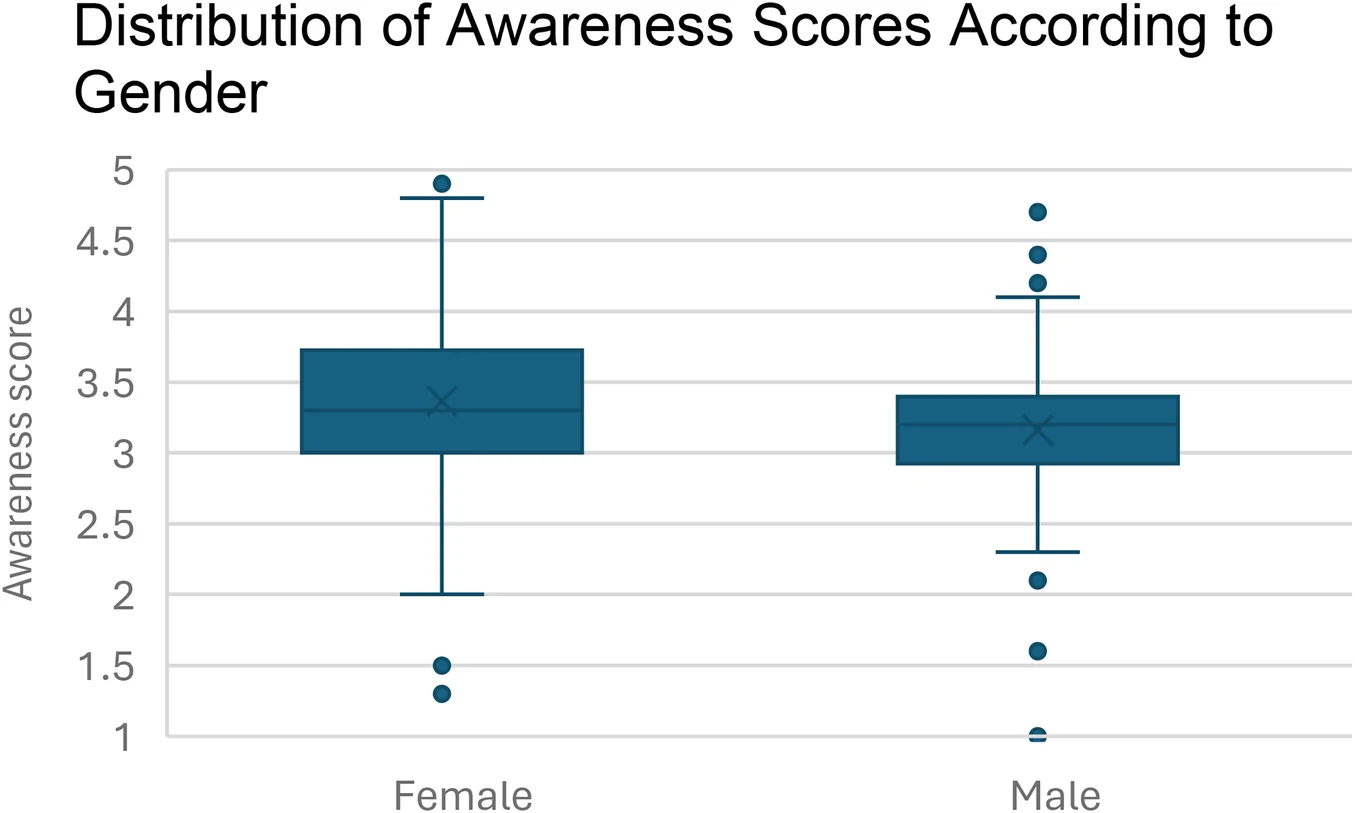
Distribution of awareness scores according to gender among the study participants (*N* = 294).

**Figure 3:**
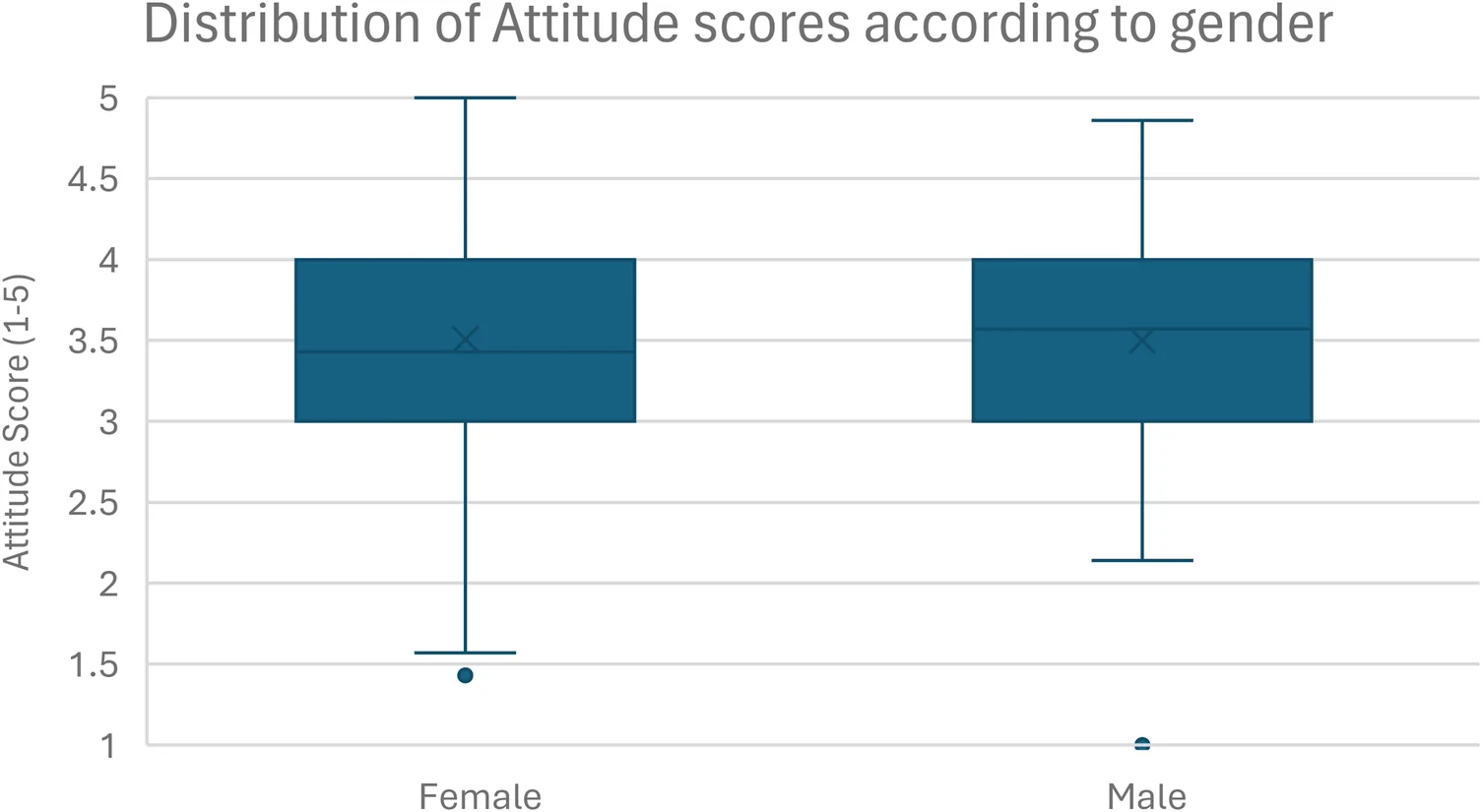
Distribution of attitude scores according to gender among the study participants (*N* = 294).

**Table 5:** Comparison of awareness and attitude scores according to gender.

Outcome	Male mean rank	Female mean rank	U	Z	*P*-Value	Effect size (R,B)
Awareness score	132.86	157.04	8,625.50	−2.392	0.017	0.165
Attitude score	151.29	145.03	9,884.50	−0.621	0.535	0.042

Values are presented as mean ranks. Comparisons between gender groups were performed using the Mann–Whitney *U*-test. Effect size was calculated using the rank-biserial correlation (rᵣb). A two-sided *p*-value < 0.05 was considered statistically significant.

### 3.6 Comparison of awareness and attitude scores according to educational status

Awareness scores differed significantly across educational groups (Kruskal–Wallis H = 17.183, df = 4, *ε*² = 0.045, *p* = 0.002; Figure 4). Bonferroni-adjusted pairwise comparisons demonstrated significant differences between participants with no formal education and those with intermediate/PUC (adjusted *p* = 0.044), graduate (adjusted *p* = 0.004), and postgraduate education (adjusted *p* = 0.028). Attitude scores did not differ significantly across educational groups (H = 9.302, df = 4, *ε*^2^ = 0.018, *p* = 0.054; Figure 5). Therefore, no *post-hoc* comparisons were performed for attitude (Table 6).

**Figure 4:**
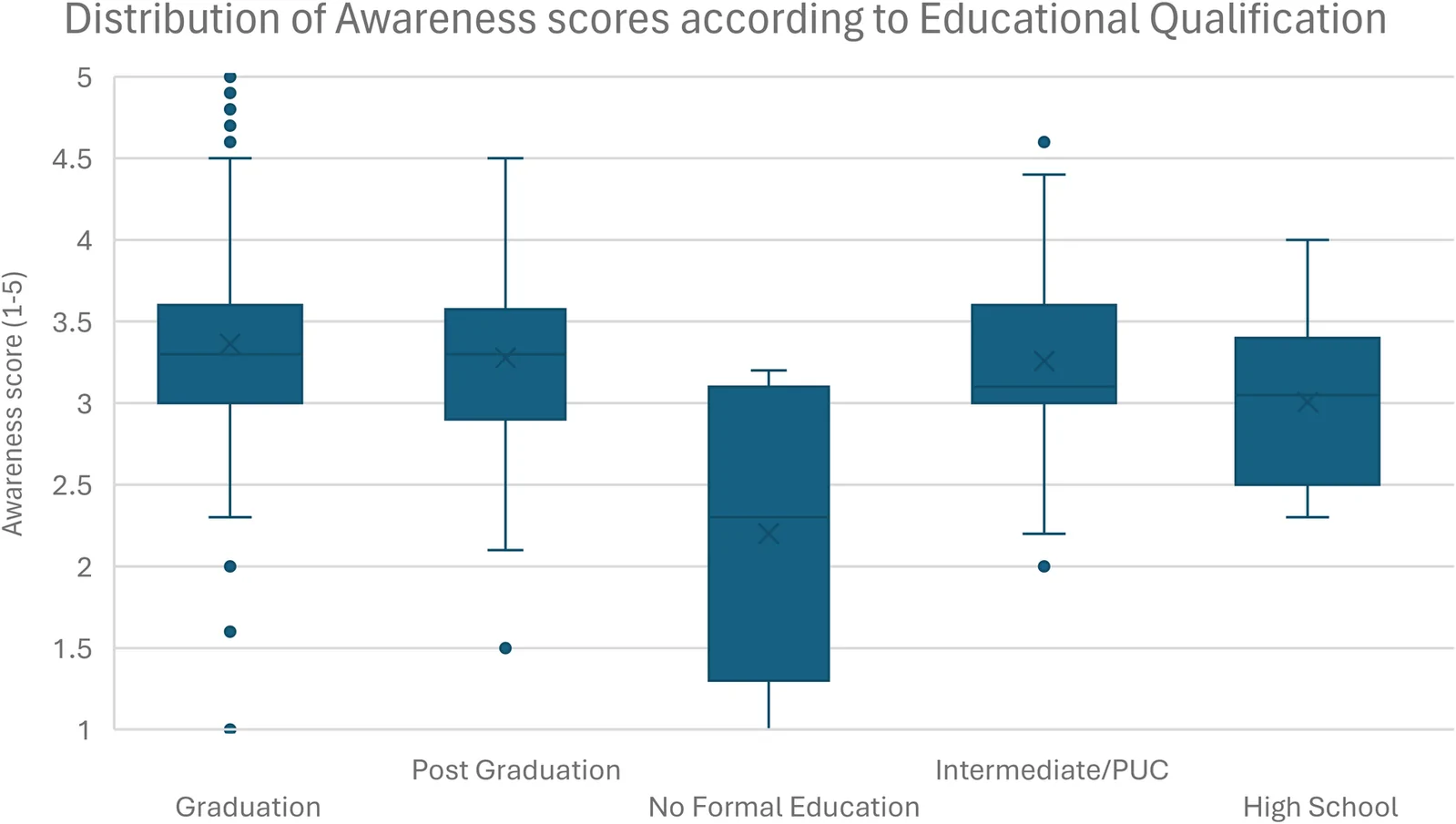
Distribution of awareness scores according to educational status among the study participants (*N* = 294).

**Figure 5:**
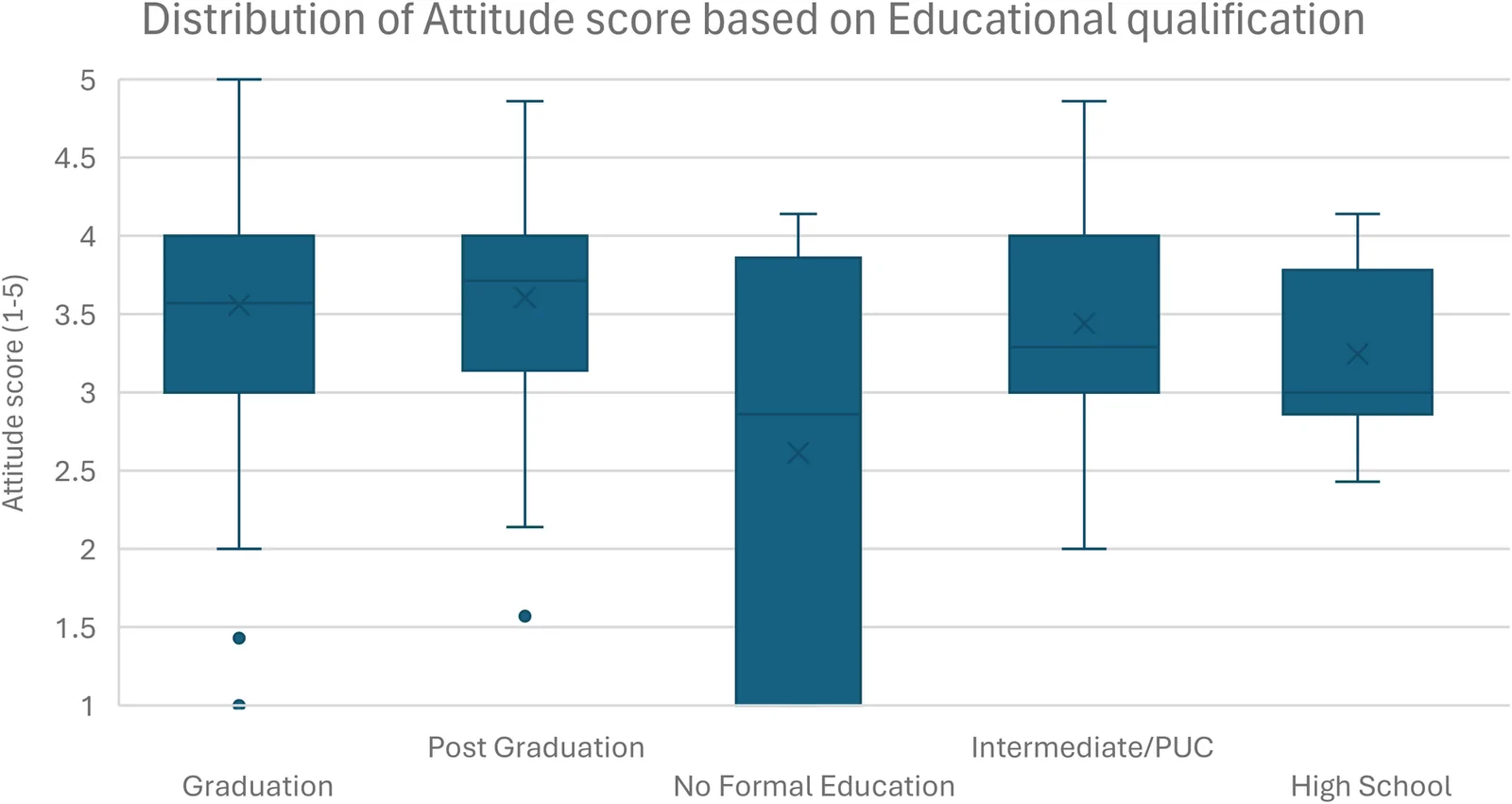
Distribution of attitude scores according to educational status among the study participants (*N* = 294).

**Table 6:** Comparison of awareness and attitude scores according to educational status.

Outcome	H	DF	*P*-VALUE	Effect size (*Ε*²)
Awareness score	17.183	4	0.002	0.045
Attitude score	9.302	4	0.054	0.018

Comparisons across educational groups were performed using the Kruskal–Wallis *H*-test. Effect size was estimated using epsilon-squared (*ε*^2^). A two-sided *p*-value < 0.05 was considered statistically significant).

### 3.7 Association between age and awareness and attitude scores

Age was not significantly correlated with awareness score (Spearman's *ρ* = 0.024, *p* = 0.680). However, age demonstrated a weak positive correlation with attitude score (Spearman's *ρ* = 0.122, *p* = 0.036).

### 3.8 Association between previous DIY use and awareness and attitude scores

Awareness scores did not differ significantly according to previous DIY use (Mann–Whitney U = 9,820.50, rᵣb ≈ 0.046, *p* = 0.506). In contrast, attitude scores differed significantly according to previous DIY use (Mann–Whitney U = 11,938.50, Z = 2.328, rᵣb ≈ 0.160, *p* = 0.020), indicating that prior experience with DIY dental practices was associated with attitude scores.

### 3.9 Correlation between awareness and attitude scores

A positive and statistically significant correlation was observed between the awareness and attitude scores (Spearman's *ρ* = 0.624, *p* < 0.001; *n* = 294), indicating that higher awareness scores were associated with higher attitude scores.

### 3.10 Multivariable logistic regression analysis

A multivariable binary logistic regression analysis was performed to examine factors associated with reported previous DIY dental practice use after adjustment for age, gender, educational status, awareness score, and attitude score. The overall model was statistically significant (*χ*^2^ = 71.615, df = 8, *p* < 0.001), with a Nagelkerke R^2^ of 0.293. Age was significantly associated with previous DIY use (adjusted OR = 1.055, 95% CI: 1.029–1.081, *p* < 0.001). Male participants had higher odds of reported previous DIY use compared with females (adjusted OR = 4.259, 95% CI: 2.442–7.428, *p* < 0.001). Educational status was significantly associated with previous DIY use overall (*p* = 0.016). Compared with postgraduate participants, participants with high-school education (adjusted OR = 0.228, 95% CI: 0.053–0.988, *p* = 0.048) and intermediate/PUC education (adjusted OR = 0.239, 95% CI: 0.091–0.627, *p* = 0.004) had lower odds of reported DIY use. Awareness score was not significantly associated with previous DIY use (adjusted OR = 0.607, 95% CI: 0.329–1.120, *p* = 0.110), while attitude score showed a borderline association that did not reach conventional statistical significance (adjusted OR = 1.655, 95% CI: 0.995–2.750, *p* = 0.052). The Hosmer–Lemeshow test was non-significant (*χ*^2^ = 13.186, df = 8, *p* = 0.106), indicating adequate model fit, and the model correctly classified 75.2% of participants. No substantial multicollinearity was identified among the predictors, with VIF values ranging from 1.025 to 1.973 (Figure 6; Table 7).

**Figure 6:**
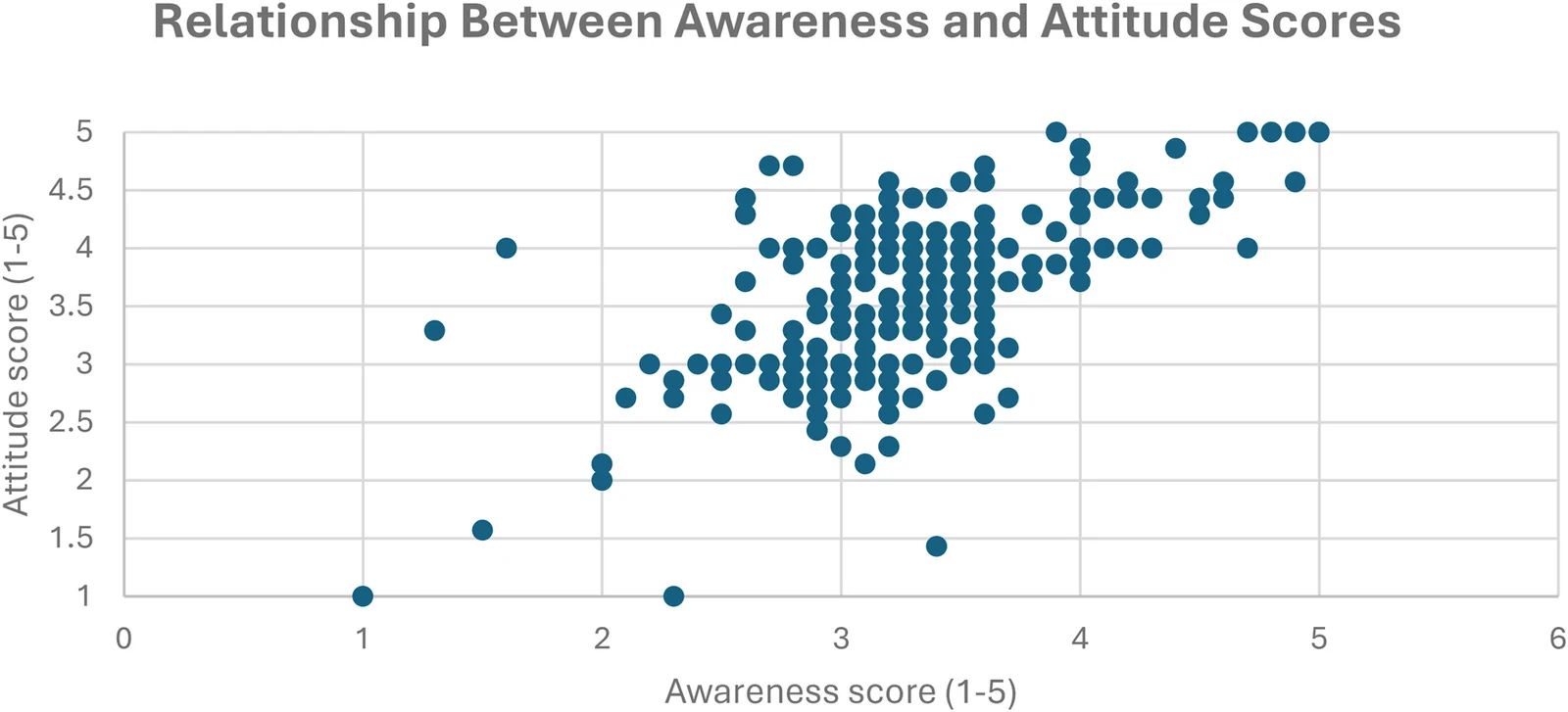
Relationship between awareness and attitude scores among the study participants (*N* = 294).

**Table 7:** Multivariable logistic regression analysis of factors associated with previous use of DIY dental practices.

Predictor	Adjusted or	95% CI	*P*-value
Awareness score	0.607	0.329–1.120	0.110
Attitude score	1.655	0.995–2.750	0.052
Gender			
Male vs. female	4.259	2.442–7.428	<0.001
Educational status			
No formal education vs. postgraduate	1.124	0.184–6.876	0.900
High school vs. postgraduate	0.228	0.053–0.988	0.048
Intermediate/puc vs. postgraduate	0.239	0.091–0.627	0.004
Graduate vs. postgraduate	0.658	0.303–1.427	0.289
Age (years)	1.055	1.029–1.081	<0.001

Adjusted odds ratios (ORs) and 95% confidence intervals (CIs) were obtained from multivariable binary logistic regression. The dependent variable was previous DIY dental practice use (No = 0, Yes = 1). Female and postgraduate education were used as reference categories.

## 4 Discussion

The increasing availability of dental information, products, and treatment options through digital platforms has made self-directed oral healthcare increasingly accessible. However, accessibility does not necessarily imply that individuals can distinguish between appropriate home care and interventions that require professional assessment. The present study examined awareness, attitudes, and reported use of DIY dental practices together, thereby providing a broader behavioural perspective than examining individual forms of self-treatment in isolation. The findings indicate that DIY dental practices are relatively common in this study population, while awareness and attitudes do not necessarily translate directly into behavioural choices.

The 39.1% prevalence of reported previous DIY dental practice use (95% CI: 33.5%–44.8%) indicates that self-directed dental care represents a relevant oral health behaviour among the participants. Among those reporting previous DIY use, aligners were the most frequently reported practice (63.5%), followed by home remedies (27.8%), whitening kits/products (11.3%), and oral hygiene products (6.1%). The predominance of aligner use is particularly relevant in view of the increasing availability of direct-to-consumer orthodontic products. Previous reports have highlighted the potential risks associated with unsupervised dental interventions, including inappropriate treatment, enamel or periodontal damage, and delayed professional care (4, 7). Katebi et al. (12) similarly reported the widespread practice of self-treatment for oral conditions, particularly where access to professional dental care may be limited. The present findings therefore suggest that contemporary DIY dentistry extends beyond traditional home remedies and increasingly includes commercially available dental products and treatment modalities.

The prominence of aligner use warrants particular attention because orthodontic treatment requires assessment of occlusion, periodontal status, dental movement, and other clinical factors that may not be adequately evaluated through consumer-directed treatment pathways. Parkar et al. (8) emphasized the importance of professional expertise and supervision when applying clinically developed dental techniques. Thus, the concern with DIY orthodontic treatment is not simply the use of a dental product without professional supervision, but the possibility that the apparent simplicity of such products may obscure the clinical assessment required before and during treatment.

Although the overall awareness score indicated a varying level of awareness, the item-level findings showed that awareness was not uniform across different aspects of DIY dentistry. Neutral responses were common for several statements, particularly those related to home remedies, social media influence, and the safety or acceptability of specific DIY practices. This suggests that participants may recognize the general possibility of harm while remaining uncertain about the safety of particular practices. However, social media exposure was not directly or quantitatively assessed in the present study; therefore, the role of social media should not be interpreted as an independently measured determinant of DIY dental practices. Harris (10) reported difficulties in distinguishing reliable oral health information from misinformation obtained online, while Baik et al. (2) identified social media as an important source of oral health information despite concerns regarding the scientific quality of some available content. Roark (3) similarly highlighted how simplified online demonstrations may portray complex dental procedures as relatively easy alternatives to professional treatment. These findings indicate that increasing the volume of oral health information alone may be insufficient; the ability to critically evaluate the reliability and clinical relevance of digital information is also important. The generally favourable attitudes toward professional dental care and stronger regulation of DIY practices observed in the present study are encouraging. Participants generally supported professional consultation and public awareness initiatives and considered professional dental care worthwhile. However, the presence of neutral or favourable responses toward selected DIY-related statements indicates that positive attitudes toward professional care do not necessarily eliminate the appeal of self-directed dental practices. Asokan et al. (13) demonstrated that although certain traditional oral health practices may have adjunctive benefits under appropriate conditions, they should not be regarded as substitutes for professional dental care. Similarly, Parkar et al. (8) highlighted the distinction between professionally supervised clinical innovation and unsupervised application by consumers. Therefore, public health interventions should acknowledge the perceived convenience and accessibility of DIY practices while clearly communicating when professional assessment is necessary.

Female participants demonstrated significantly higher awareness scores than males, whereas attitude scores did not differ significantly by gender. Although the difference in awareness by gender was statistically significant, its magnitude was small (rank-biserial correlation = 0.165), indicating limited practical importance. In the multivariable logistic regression analysis, age and gender were significantly associated with reported previous DIY dental practice use after adjustment for the other variables in the model. Older age was associated with higher odds of reported previous DIY use, while male participants had higher odds compared with female participants. Educational status was also significantly associated with reported previous DIY use overall. Awareness score was not significantly associated with previous DIY use, whereas attitude score showed a borderline, non-significant association. These findings indicate that demographic characteristics may be associated with reported DIY dental practice use, while the relationship between awareness, attitudes, and behaviour appears more complex. Given the cross-sectional design, these associations should not be interpreted as causal or as evidence that awareness or attitude preceded previous DIY use. Previous literature has identified financial considerations, accessibility, and perceptions of professional dental care as potential influences on self-directed treatment behaviours (11, 14, 15). However, these factors were not comprehensively measured in the present study and therefore should be considered potential explanations rather than established determinants.

Educational status was significantly associated with awareness, with Bonferroni-adjusted comparisons demonstrating differences particularly between participants with no formal education and those with intermediate/PUC, graduate, and postgraduate education. This finding is consistent with the broader importance of educational attainment in understanding and interpreting health information. AlQuahthani et al. (15) similarly reported that inadequate awareness of the risks associated with self-medication may contribute to unsafe oral health practices. However, the magnitude of the educational difference was small (*ε*^2^ = 0.045), indicating limited practical importance despite statistical significance. Attitude scores did not differ significantly across educational groups (*p* = 0.054). Therefore, the present findings do not support the interpretation that higher educational attainment necessarily results in more favourable attitudes toward professional dental care. Rather, education may influence recognition of risks without producing a corresponding difference in attitudes or behaviour.

Previous DIY use was not significantly associated with awareness scores, whereas attitude scores differed significantly according to previous DIY use. This finding is important because it suggests that individuals who had previously engaged in DIY dental practices did not necessarily possess lower awareness of associated risks. Instead, differences were observed in attitudes. This distinction between knowledge of risk and behavioural decision-making is relevant when designing interventions. Financial constraints and limited access to dental care have previously been identified as factors encouraging self-treatment or delayed professional consultation (14, 16). Nevertheless, because the present study is cross-sectional, it cannot determine whether particular attitudes preceded DIY use or were influenced by previous experience.

The positive correlation between awareness and attitude scores (Spearman's *ρ* = 0.624, *p* < 0.001) indicates that participants with higher awareness scores also tended to report higher attitude scores. However, some items included in the awareness measure had perception- or attitude-related content, including preferences for professional consultation, confidence in home remedies, and perceived affordability of DIY dental options. Therefore, the observed correlation may partly reflect conceptual overlap between the two measures. The association should consequently be interpreted cautiously, and the correlation should not be taken as evidence of a clearly distinct relationship between awareness and attitude. Furthermore, the cross-sectional design does not permit conclusions regarding the direction of this association. The findings have important implications for dental public health. The relatively high prevalence of reported DIY use, particularly the predominance of aligner use, highlights the need for targeted communication regarding the limitations and potential risks of unsupervised dental interventions. Dental professionals and public health practitioners should actively contribute to evidence-based digital oral health communication, particularly through platforms where consumers obtain dental information and recommendations. Digital interventions should focus not only on providing information but also on helping individuals identify unreliable claims, recognize potential warning signs, and understand when professional assessment is required.

Regulatory and professional measures may complement these educational approaches. Monitoring commercially promoted DIY dental products and misleading online advertisements may help reduce unrealistic expectations regarding the safety and effectiveness of unsupervised dental procedures. Similar guidance may help distinguish appropriate home-based oral care from potentially harmful self-treatment.

### 4.1 Strengths and limitations

A major strength of the present study is its multidimensional assessment of DIY dentistry, incorporating awareness, attitudes, reported use, and the types of DIY practices undertaken, together with their relationships with demographic characteristics. The study also retained individual questionnaire items outside the composite scales, allowing areas of uncertainty to be identified rather than relying solely on overall scores. Furthermore, the use of appropriate non-parametric analyses, prevalence estimation with a confidence interval, correlation analysis, and multivariable logistic regression provided complementary perspectives on the study objectives.

However, several limitations should be acknowledged. First, the cross-sectional design prevents conclusions regarding temporal or causal relationships between awareness, attitudes, and DIY use. Second, convenience sampling and recruitment from a single department of a dental college limit external validity. Participants attending a dental institution may differ from individuals who do not seek professional dental care; therefore, the observed prevalence should not be interpreted as representative of the wider Indian population. Selection bias may have resulted in an over- or under-estimation of the prevalence of DIY dental practices because participants seeking dental care may differ systematically from individuals who do not seek professional care; the direction and magnitude of this potential bias cannot be determined from the present data Third, the study relied on self-reported previous DIY practices and was therefore susceptible to recall and social desirability biases. Although internal consistency of the awareness and attitude scales was assessed using Cronbach's alpha, formal construct validation through exploratory or confirmatory factor analysis and test–retest reliability assessment were not performed. Some questionnaire items may conceptually overlap across the awareness and attitude domains, including items related to professional consultation, confidence in home remedies, perceived affordability of DIY dental options, and regulation of DIY dental practices. This conceptual overlap may have contributed to the observed correlation between awareness and attitude scores and limits the extent to which these measures can be interpreted as entirely distinct constructs. Fourth, the no-formal-education category contained only seven participants (2.4%), resulting in an imprecise regression estimate (adjusted OR = 1.124; 95% CI: 0.184–6.876). This subgroup was retained as a distinct category to preserve the original educational classification, but the corresponding estimate should be interpreted with caution.

Finally, potentially relevant behavioural and contextual factors, including income and other socioeconomic characteristics, rural/urban residence, dental insurance coverage, frequency of dental visits, dental anxiety, perceived affordability, accessibility of dental services, and exposure to social media content, were not comprehensively assessed. These unmeasured factors may have influenced reported DIY use and may therefore represent potential sources of residual confounding. Future multicentre, community-based studies using more representative sampling methods are required to estimate population-level prevalence and investigate the broader determinants of DIY dental practices.

### 4.2 Implications for future research

Future research should move beyond assessing whether individuals are aware of the risks of DIY dentistry and investigate why individuals may continue to engage in DIY practices despite awareness of potential harm. Qualitative and mixed-methods studies could explore the influence of cost, convenience, dental anxiety, previous dental experiences, accessibility of services, social media exposure, and trust in online information. Intervention studies could subsequently assess whether digital oral health literacy programmes, professional-led social media communication, and appropriate regulation of direct-to-consumer dental products can reduce potentially unsafe DIY dental practices.

## 5 Conclusion

The present study demonstrates that DIY dental practices constitute a relevant oral health behaviour in the study population, with 39.1% reporting previous use, and that aligners were the most frequently reported practice. “Participants demonstrated varying levels of awareness regarding the risks and potential consequences of DIY dental practices. The findings also indicate that awareness, attitudes, and behaviour do not operate in a simple linear manner. Strengthening digital oral health literacy, evidence-based communication, access to professional dental care, and appropriate oversight of consumer-directed dental products may therefore provide a more comprehensive public health approach to reducing potentially unsafe DIY dental practices.

## Data Availability

The original contributions presented in the study are included in the article/Supplementary Material, further inquiries can be directed to the corresponding author.
