# Supplementary material for: Awareness and attitudes towards risks of do-it-yourself dental practices among adults attending a dental college in Mangalore city, Karnataka: a cross-sectional study
Source: Front Oral Health. 2026 Oct 29;7:1945086. doi: 10.3389/froh.2026.1945086 (PMC13760349; doi:10.3389/froh.2026.1945086)
Supplement: Supplementary file 1 [file Datasheet1.pdf]

AWARENESS AND ATTITUDES TOWARD RISKS OF “DO-IT-YOURSELF” DENTAL PRACTICES: A QUESTIONNAIRE STUDY

* Indicates required question

Participant Information Sheet And Informed consent

Awareness and attitudes toward risks of “do-it-yourself”
Dental practices: a questionnaire study
Version 3 26-12-2025
Page 1 of 9

PARTICIPANT INFORMATION SHEET
(ENGLISH)
Title of the study: AWARENESS AND ATTITUDES TOWARDS RISKS OF 'DO-IT-YOURSELF' DENTAL PRACTICES: A QUESTIONNAIRE-BASED STUDY
Details of the Investigators:
Dr. Shaik Saaffaath Muzammil, PG Student, Department of Public Health Dentistry, YDC.
Dr. Junaid, Reader, Department of Public Health Dentistry, Yenepoya Dental College, Deralakatte,
Mangalore.
The purpose of the study: The study is being carried out to assess the awareness and attitudes
towards risks of ‘do-it-yourself’ dental practices. The data collection will be done through a questionnaire made in Google Forms, which will be sent to the participants through WhatsApp
Messenger.
Study Setting: Yenepoya Dental College, Mangalore
Duration of the study: The process of filling up the questionnaire will take about 15 to 20 minutes
of your time.
Why are you being selected?
The study is being conducted among the patients visiting the department of Public Health Dentistry
in Yenepoya Dental College; hence, you are requested to take part in the study.
The benefits of the study:
This study will help to know the current understanding the participant’s awareness and attitudes
towards risks of ‘do-it-yourself’ dental practices. This will help us identify the sources which are
influencing the people to practice “do-it-yourself” dental treatments. As soon as the participant fills
the questionnaire, e poster containing risks associated with Do-It-Yourself dental procedures will be
sent through Mail to make the participants aware of Do-It-Yourself dental procedures.
Details of the intervention:
There is no intervention.
What are the risks/harms involved in the study?
No risk for participants in this study.
What are the responsibilities of the participant?
You need to read the participant information sheet and understand about the study. For any queries
you may contact Dr.Shaik Saaffaath Muzammil, 9381066758. If you consent and wish to participate
in the study, then you need to click the link provided in the google form, following which a new web

page will open with the study questionnaire. You are expected to answer the questions and click
submit button.
Awareness and attitudes toward risks of “do-it-yourself”
Dental practices: a questionnaire study
Version 3 26-12-2025
Page 2 of 9

Rights of the participant: The enrolment for the study is voluntary. You may withdraw from the
study at any point of time and this decision will not affect your work in any way. You may ask your
doubt to the investigator at any point of time.
Compensation for study participation: No monetary or any other benefits will be provided.
Privacy and Confidentiality of the participants: Your privacy will be strictly protected throughout
the study. You will not be asked to provide your name or any personal identifiers in the questionnaire.
Your phone number will be collected only for the purpose of sending the questionnaire link and will
not be linked to your responses. All responses will be anonymized and stored securely in a password
protected system accessible only to the research team. The data will be used solely for academic and
research purposes and will be reported only in aggregated form without revealing individual identities
Reporting and publication of the research: The findings of the study are intended to be published
in a peer-reviewed indexed journal. Only group data will be presented in the results and not individual
data. A report of the study will be submitted to the ethics committee.
Whom can you approach for clarifications?
For clarifications regarding the study, you can approach :
Dr Shaik Saaffaath Muzammil (1st Year MDS Student)
Phone no: 9381066758
Mail id: saaffushaik07@gmail.com
Dr. Junaid
Reader
Department of Public Health Dentistry
Yenepoya Dental college,
Deralakatte, Mangalore.
Ph.no. 9964012940
Mail Id: junaidbantwal@yenepoya.edu.in
Details of a responsible member of the ethics committee who will address the concerns on the rights
of the research participant, in case he/she is not satisfied by the responses from the PI:
ETHICS COMMITTEE:
Dr. Leena Pramod,
Member secretary,
Yenepoya Ethics Committee – 2 -2 Basement,
Yenepoya Dental College.
Email: yec2@yenepoya.edu.in

Contact no- 0824-2206000.

PARTICIPANT INFORMATION SHEET (KANNADA)

ಇಂದಿನ ಅಧ್ಯಯನದ ಶೀರ್ಷಿಕೆ:
'ಡೂ-ಇಟ್-ಯುರ್ಸೆಲ್ಫ್ ' ದಂತ ಚಿಕಿತ್ಸೆ ಗಳ ಅಪಾಯಗಳ ಬಗ್ಗೆ ಜಾಗೃತಿ ಮತ್ತು
ವಲ್ನ ೊೀಟಗಳು: ಪ್ರಶ್ನನವಳಿ ಆಧಾರಿತ ಅಧ್ಯಯನ.
ಅನ್ವ ೀಷಕರ ವಿವರಗಳು:
ಡಾ. ಶೈಕ್ ಸಾಫಾತ್ ಮುಜಮ್ಮಿಲ್ಪ, ಪಿಜಿ ವಿದ್ಯಯರ್ಥಿ, ಸಾವೆಜನಿಕ ಆರೋಗಯ ದಂತಶ್ನಸ್ತು ರ
ವಿಭಾಗ, ವೈಡಿಸಿ. ಡಾ. ಜುನ್ಯದ್, ರೋಡರ್, ಸಾವೆಜನಿಕ ಆರೋಗಯ ದಂತಶ್ನಸ್ತು ರ ವಿಭಾಗ,
ಯೆನೋಪಾಯ ದಂತ ಕಾಲೋಜು, ದೋರಳಕಟ್ಟೆ , ಮಂಗಳೂರು.
ಅಧ್ಯಯನದ ಉದ್ದ ೀಶ:
ಈ ಅಧ್ಯಯನವು ‘ಡೂ-ಇಟ್-ಯುರ್ಸೆಲ್ಫ್ ’ ದಂತ ಚಿಕಿತ್ಸೆ ಗಳ ಅಪಾಯಗಳ ಬಗ್ಗೆ ಜನರ
ಜಾಗೃತಿ ಮತ್ತು ವಲ್ನ ೊೀಟಗಳನ್ನನ ಅಳೆಯುವ ಉದದ ೊೀಶದಂದ ಕೈಗೊಳಳಲಾಗುತಿ ದ.
ಡೋಟಾ
ಸ್ತಂಗರಹಣೆಗಾಗಿ ಗೂಗಲ್ಪ ಫಾರ್ಮೆನಲ್ಲಿ ರೂಪಿಸಿದ ಪ್ರಶ್ನನವಳಿಯನ್ನನ ವಾಟಸೆಪ್
ಮೆಸ್ತಂಜರ್ ಮೂಲಕ ಪಾಲುಗೊಳುಳವವರಿಗ್ಗ ಕಳುಹಿಸ್ತಲಾಗುತು ದ.
ಅಧ್ಯಯನ ಸ್ಥಳ:
ಯೆನೋಪಾಯ ದಂತ ಕಾಲೋಜು, ಮಂಗಳೂರು
ಅಧ್ಯಯನ ಅವಧಿ:
ಪ್ರಶ್ನನವಳಿಯನ್ನನ ಭತಿೆ ಮಾಡುವ ಪ್ರ ಕಿರಯೆಗ್ಗ ನಿಮಿ 15 ರಿಂದ 20 ನಿಮ್ಮಷಗಳು
ಬೋಕಾಗಬಹುದು.
ನೀವು ಏಕೆ ಆಯ್ಕೆಯಾಗಿದಿದ ೀರಿ?
ಈ ಅಧ್ಯಯನವನ್ನನ ಯೆನೋಪಾಯ ದಂತ ಕಾಲೋಜಿನ ಸಾವೆಜನಿಕ ಆರೋಗಯ ದಂತಶ್ನಸ್ತು
ರ
ವಿಭಾಗಕೆ ಭೋಟಿ ನೋಡುವ ರೋಗಿಗಳಲ್ಲಿ ನಡಸ್ತಲಾಗುತಿ ದ; ಆದದರಿಂದ ನಿಮಿನ್ನನ
ಭಾಗವಹಿಸ್ತಲು ವಿನಂತಿಸ್ತಲಾಗುತಿ ದ.
ಅಧ್ಯಯನದ ಲಾಭಗಳು:
ಈ ಅಧ್ಯಯನದ ಮೂಲಕ ‘ಡೂ-ಇಟ್-ಯುರ್ಸೆಲ್ಫ್ ’ ದಂತ ಚಿಕಿತ್ಸೆ ಗಳ ಅಪಾಯಗಳ ಕುರಿತ್ತ
ಪಾಲುಗೊಳುಳವವರ ಜಾಗೃತಿ ಮತ್ತು ವಲ್ನ ೊೀಟಗಳ ನ್ಯಜ ಸಿತಿಯನ್ನನ
ತಿಳಿದುಕೊಳಳಬಹುದ್ಯಗಿದ. ಈ ಮೂಲಕ ಜನರು ಇಂತಹ ಸ್ತವಯಂ-ಚಿಕಿತ್ಸೆ ಗಳಿಗ್ಗ ಪ್ರ ೊೀರಣೆ
ಪ್ಡಯುತಿರುವ ಮೂಲಗಳನ್ನನ ಗುರುತಿಸ್ತಬಹುದು. ಭಾಗವಹಿಸುವವರು ಪ್ರಶ್ನನವಳಿ

ಭತಿೆಯನ್ನನದ ಮೋಲ, 'ಸ್ತವಯಂ
ಮಾಡಿಕೊಳುಳವ ದಂತ ಚಿಕಿತ್ಸೆ ಗಳ ಅಪಾಯಗಳು' ಎಂಬ ವಿಷಯದ ಬಗ್ಗೆ
ಜಾಗೃತಿಯನ್ನನ ಂಟುಮಾಡಲು ಸ್ತಂಬಂಧಿಸಿದ ಇ-ಪೋಸ್ತೆರ್ ಅನ್ನನ ಇಮೋಲ್ಪ ಮೂಲಕ
ಕಳುಹಿಸ್ತಲಾಗುತು ದ.

ಹಸ್ತ ಕೆಷ ೀಪದ ವಿವರಗಳು:
ಈ ಅಧ್ಯಯನದಲ್ಲಿ ಯಾವುದೋ ರೋತಿಯ ಹಸ್ತು ಕಷ ೊೀಪ್ವಿಲಿ .
ಅಧ್ಯಯನದಲ್ಲಿ ಭಾಗವಹಿಸುವ ಅಪಾಯಗಳು/ಹಾನ:
ಈ ಅಧ್ಯಯನದಲ್ಲಿ ಪಾಲುಗೊಳುಳವವರಿಗ್ಗ ಯಾವುದೋ ಅಪಾಯವಿಲಿ .
ಪಾಲುಗೊಳುವವರ ಹೊಣೆಗಾರಿಕೆಗಳು:

ನೀೋವು ಭಾಗವಹಿಸುವ ಮೊದಲು, ಭಾಗವಹಿಸುವವರ ಮಾಹಿತಿಪತ್ರವನ್ನುನ ಒದ ಅಧ್ಯಯನದ ಬಗ್ಗೆ ತಿಳಿದುಕೊಳ್ಳಬೋಕು. ಯಾವುದೋ ಪ್ರ ಶನ ಗಳಿದದರೆ, ಡಾ. ಶೈಕ್ ಸಾಫಾತ್ ಮುಜಮ್ಮಿಲ್ಪ ಅವರನ್ನುನ 9381066758 ಸ್ತಂಖ್ಯೆಯಲ್ಲಿ ಸ್ತಂಪ್ಕಿಸ್ತಬಹುದು. ನೀೋವು ಅಧ್ಯಯನದಲ್ಲಿ ಪಾಲ್ಗೆಳ್ಳಲು ಒಪಿಕೊಂಡರೆ, ಗೂಗಲ್ಪ ಫಾರ್ಮಿನ ಲ್ಲಿಂಕ್ ಮೋಲ ಕಿಕ್ ಮಾಡಬೋಕು. ನಂತರ ಹೊಸ್ತ ವಬಮಪ್ೇಜ್ ತ್ಸರೆಯಲ್ಲದ, ಅಲ್ಲಿ ಪ್ರಶ್ನೆನವಳಿಯನ್ನುನ ಭತಿ ಮಾಡಿ 'ಸ್ತಬ್ಮಿಟ್' ಬಟನ್ ಒತುಬೋಕು.

ಪಾಲುಗೊಳುುವವರ ಹಕ್ಕುಗಳು:

ಈ ಅಧ್ಯಯನದಲ್ಲಿ ಭಾಗವಹಿಸುವುದು ಪೂರ್ಣವಾಗಿ ಐಚಿ ಕ. ನೀೋವು ಯಾವುದೋ ಸ್ತಮಯದಲ್ಲಿಯೋ ಅಧ್ಯಯನದಂದ ಹಿಮಿಟೆಬಹುದು ಮತ್ತು ಇದರಿಂದ ನಿಮಿ ಕಾಯೆದಲ್ಲಿ ಯಾವುದೋ ಪ್ರಿಣಾಮ ಉಂಟಾಗುವುದಲಿ . ನೀೋವು ಯಾವುದೋ ಸ್ತಮಯದಲ್ಲಿಯೋ ಸ್ತಂಶೋಧ್ಕರಿಗ್ಗ ನಿಮಿ ಸ್ತಂದೋಹವನ್ನುನ ಕೋಳಬಹುದು.

ಪಾಲುಗೊಳುುವವರಿಗೆ ನೀಡುವ ಪರಿಹಾರ:

ಈ ಅಧ್ಯಯನದಲ್ಲಿ ಪಾಲ್ಗೆಳುುವಿಕಗಾಗಿ ಹಕ್ಷಾಸು ಅಥವಾ ಯಾವುದೋ ಇತರ ರೋತಿಯ ಲಾಭ ನೀೋಡಲಾಗುವುದಲಿ .

ಪಾಲುಗೊಳುುವವರ ಗೌಪಯತೆ:

ಈ ಅಧ್ಯಯನದ ದ್ಯಖಲಾತಿಗಳನ್ನುನ ಖಾಸ್ತಗಿ ರೋತಿಯಲ್ಲಿ ಇಡಲಾಗುವುದು. ಎಲಿ ಮಾಹಿತಿಯನ್ನುನ ಗುಪ್ತವಾಗಿರಿಸ್ತಲಾಗುತು ದ. ಯಾವುದೋ ವರದಯಲ್ಲಿ ನಿಮಿನ್ನುನ ಗುರುತಿಸ್ತಬಹುದ್ಯದ ಮಾಹಿತಿಯನ್ನುನ ನಮೂದಸ್ತದು. ನಿಮಿ ಹೆಸ್ತರು ಅಥವಾ ದೂರವಾಣಿ ಸ್ತಂಖ್ಯೆಯ ಕೋಳಲಾಗುವುದಲಿ . ನಿಮಿ ಹೆಸ್ತರು ಮತ್ತು ಗುರುತಿಸುವ ಮಾಹಿತಿಯನ್ನುನ ಈ

ಸ್ತಮೋಕಷಯಂದಗ್ಗ ಸ್ತಂಗರಹಿಸ್ತಲಾಗುವುದಲಿ . ಸ್ತಮೋಕಷ ಗಳನ್ನುನ ಲಾಕ್ ಮಾಡಲಾದ ಫೈಲ್ಪಮನಲ್ಲಿ ಇಡಲಾಗುವುದು; ಸ್ತಂಶೋಧ್ನ್ನ ತಂಡದ ಸ್ತದಸ್ತಯರು, ನೈತಿಕ ಸ್ತಮ್ಮತಿ ಮತ್ತು ನಿಯಂತರಣಾಧಿಕಾರಿಗಳಿಗಷ್ಟೆ ದ್ಯಖಲಾತಿಗಳಿಗ್ಗ ಪ್ರವೋಶವಿರುತು ದ.

ಅಧ್ಯಯನ ವರದಿ ಮತ್ತತ ಪರ ಕಟಣೆ:

ಅಧ್ಯಯನದ ಫಲ್ಲತಂಶವನ್ನುನ ಪಿಯರ್-ರಿವ್ಯುಯ ಆಗಿರುವ ಇಂಡಕ್ಸ್ ಜನೆಲ್ಪಮನಲ್ಲಿ ಪ್ರ ಕಟಿಸುವ ಉದದ ೇಶವಿದ. ಫಲ್ಲತಂಶಗಳಲ್ಲಿ ಗುಂಪುಮಟೆದ ಡೋಟಾವನ್ನುನ ಮಾತರ ನೀೋಡಲಾಗುತು ದ, ವೈಯಕಿು ಕ ಮಾಹಿತಿಯನ್ನುನ ಸೋರ್ರಿಸ್ತಲಾಗದು. ಅಧ್ಯಯನದ ವರದಯನ್ನುನ

ನೈತಿಕ ಸ್ತಮ್ಮತಿಗ್ಗ ಸ್ತಲ್ಲಿ ಸ್ತಲಾಗುವುದು.

ಸ್ಂಶಯಗಳಿಗಾಗಿ ಸ್ಂಪರ್ಕಿಸ್ಬಹುದಾದವರು:

ಡಾ. ಶೈಕ್ ಸಾಫಾತ್ ಮುಜಮ್ಮಿಲ್ಪ (1ನೋ ವಷೆ ಎಂ.ಡಿಎಸ್ ವಿದ್ಯಯರ್ಥಿ)
ದೂರವಾಣಿ: 9381066758
ಇಮೋಲ್ಪ: saaffushaik07@gmail.com

ಡಾ. ಜುನೈದ್
ರೋಡರ್
ಸಾವೆಜನಿಕ ಆರೋಗಯ ದಂತಶ್ನಸ್ತು ರ ವಿಭಾಗ
ಯೆನೋಪಾಯ ದಂತ ಕಾಲೋಜು, ದೋರಳಕಟ್ಟೆ , ಮಂಗಳೂರು
ದೂರವಾಣಿ: 9964012940
ಇಮೋಲ್ಪ: dr.junaid.bantwal@gmail.com

ಪಾಲುಗೊಳುುವವರ ಹಕ್ಕುಗಳ ಬಗೆ ನಮಗೆ ಇನನಷ್ಟು ಮಾಹಿತಿ ಬೀಕಾದಲ್ಲಿ ಅಥವಾ ಮುಖ್ಯ ಸ್ಂಶೀಧ್ಕರ ಉತ್ತ ರದಿಂದ ನೀವು ತೃಪತರಾಗದಿದದಲ್ಲಿ , ನೀವು ಈ ಕೆಳಗಿನ ನ್ೈತಿಕ ಸ್ಮಿತಿಯ ಸ್ದಸ್ಯರನ್ನುನ ಸ್ಂಪರ್ಕಿಸ್ಬಹುದು:

ನ್ೈತಿಕ ಸ್ಮಿತಿ:

ಡಾ. ಲ್ಲೋನ್ನ ಪ್ರಮೋದ್
ಮೆಂಬರ್ ಸ್ಕರಟರಿ
ಯೆನೋಪಾಯ ಎಥ್ಕ್ಸ್ ಕಮ್ಮಟಿ – 2 -2ನೋ ತಳಮಹಡಿ, ಯೆನೋಪಾಯ ದಂತ ಕಾಲೋಜು
ಇಮೋಲ್ಪ: yec2@yenepoya.edu.in
ಸ್ತಂಪ್ಕೆ ಸ್ತಂಖ್ಯೆಯ : 0824-2206000.

Awareness and attitudes toward risks of “do-it-yourself”
Dental practices: a questionnaire study
Version 3 26-12-2025
Page 6 of 9

PARTICIPANT INFORMATION SHEET (MALAYALAM)
പഠനത്തിന്റെ ശീർഷകം:
'ഡൂ-ഇറ്റ്-യ ോർസെൽഫ്' ദന്ത ചികിത്സോ രീതികളുസെ
അപകെങ്ങസളക്കുറിച്ചുള്ള യ ോധവുമും െമീപനങ്ങളും: ഒരു
യചോദയോവലി അെിസ്ഥോനത്തിലുള്ള പഠനും.
ഗവേഷകരുറെ േിശദ ംശങ്ങൾ:
യഡോ. സെ ്ഖ് േോെഫോത്ത് മുചമ്മിൽ, പി.ജി വിദയോർത്ഥി, സപോതു
ആയരോഗ്യ ദന്തവിദയ വിഭോഗ്ും, YDC.
യഡോ. ജുനനദ്, റീഡർ, സപോതു ആയരോഗ്യ ദന്തവിദയ വിഭോഗ്ും,
സ നയപോ ദന്ത യകോയളജ്, യദരലക്കസെ, മുംഗ്ളൂരു.
പഠനത്തിന്റെ ഉവേശയം:
‘ഡൂ-ഇറ്റ്-യ ോർസെൽഫ്’ ദന്ത ചികിത്സോ രീതികളുസെ
അപകെങ്ങസളക്കുറിച്ചുള്ള യ ോധവുമും െമീപനങ്ങളുമോണ് ഈ
പഠനും വില ിരുത്തുന്നത്. ഗ്ൂഗ്ിൾ യഫോമിൽ തയ്യോറോക്കി
യചോദയോവലി വോട്ട്സ ്ആപ്പ് സമെഞ്ചറു വഴി
പസെടുക്കുന്നവർക്ക ്ക്കും.
പഠനസ്ഥലം:
സ നയപോ ദന്ത യകോയളജ്, മുംഗ്ളൂരു
പഠനക ല േധി:
യചോദയോവലി പൂരിപ്പിക്കോൻ നിങ്ങളിൽ നിന്നും ഏകയദശും 15 മുതൽ
20
മിനിറ്റ് വസര െമ സമെടുക്കും.
നിങ്ങൾ എന്തുറക ണ്ട് തിരസെടുക്കസെട്ടിരിക്കുന്നു?
സപോതു ആയരോഗ്യ ദന്തവിദയ വിഭോഗ്ും െന്ദർശിക്കുന്ന
യരോഗ്ികളിലോണ്
പഠനും നെത്തുന്നത്; അതിനോൽ നിങ്ങസള പസെടുക്കോൻ

ക്ഷണിച്ചിരിക്കുന്നു.
പഠനത്തിന്റെ ഗുണങ്ങൾ:
ഈ പഠനും പസെടുത്തവരുസെ ‘ഡൂ-ഇറ്റ്-യ ോർസെൽഫ്’
ചികിത്സകസളപ്പറ്റി ുള്ള യ ോധവും െമീപനങ്ങളും മനെിലോക്കോൻ
െഹോ ിക്കും. ഇത്തരും ചികിത്സോ രീതികളിയലക്കുള്ള
യരപരണ ോ ി
രപവർത്തിക്കുന്ന ഉറവെിങ്ങൾ ഇതിലൂസെ കസെത്തോൻ േോെധിക്കും.
ഭോഗ്ങ്ങസളെുത്തവർ കവെ്ഷചസന ർ പൂരിപ്പിച്ചതിനു യശഷും, 'െവ ും
സച ുന്ന ദന്തപരിചരണ രീതി'കളുസെ അപകെങ്ങസളക്കുറിച്ചുള്ള
അവയ ോധും ഉ ർത്തുന്നതിനോ ി ഡസപ്പെ ഇ-യപോസ്റ്റർ ഇസമ ിൽ

വഴി അ ്ക്കുന്നതോ ിരിക്കും.

ഇന്െർറേൻഷൻ േശദ ംശങ്ങൾ:
ഈ പഠനത്തിൽ ഇതുവസര ഒരു ഇന്റർസവൻെനും
ഉൾസപ്പെുത്തി ിെല്ല.
പറെുക്കുന്നതിന്റെ അപകെസ ധയതകൾ:
പസെുക്കുന്നവർക്കോ ി ഈ പഠനത്തിൽ ോസതോരു അപകെവും ഇല്ല.
പറെുക്കുന്നേരുറെ ഉത്തരേ ദിത്തങ്ങൾ:
പസെുക്കുന്നതിനു മുൻപ് പൈോളി വിവര പരതും വോ ിക്കുക ും
പഠനും മനിെലോക്കുക ും സചയ്യണും. ൊംശ ങ്ങൾക്ക് യഡോ. സെ ്ഖ്
ൈോഫോത്ത് മുചമ്മിൽ (9381066758) എന്ന നമ്പറിൽ ഡസപ്പെോും.
നിങ്ങൾ െമ്മതിക്കുന്നുസവെിൽ ഗ്ൂഗ്ിൾ യഫോമിന്സറ ലെിിൽ
ക്ലിക്കുസചയ്യുക. അതനെുരിച്ച് യചോദയോവലി െഹിതും ഒരു പുതി
സവ ് യപജ് തുറ ും, അവിസെ യചോദയങ്ങൾ പൂരിപ്പിച്ച് “െബ്മിറ്റ്”
ക്ലിക്കുസചയ്യണും.
പെ ളിയുറെ അേക ശങ്ങൾ:
പഠനത്തിൽ പസെുക്കുന്നത് നെവച്ഛികമോണ്. നിങ്ങൾക്കു
െമ യമത് ആസണെിലും പഠനും ഒഴിവോക്കോും, അതിനോൽ
നിങ്ങളുസെ യജോലി/യെവനത്തിൽ ോസതോരു ോധവുമൈോകില്ല.
Awareness and attitudes toward risks of “do-it-yourself”
Dental practices: a questionnaire study
Version 3 26-12-2025
Page 8 of 9

നിങ്ങൾക്ക് നിങ്ങൾക്ക് ആവശയമുള്ളയപ്പോൾ ഗ്യവെകയരോ്
ൊംശ ങ്ങൾ യചോദിക്കോവുന്നതോണ്.
പെ ളികൾക്ക് ലഭിക്കുന്ന പ്രതിഫലം:
പസെുക്കുന്നതിന് ോസതോരു ൈോമ്പത്തികയമോ മറ്റ്
രപയതയകയമോ
ൊലഭതകൾ നൽകുന്നതല്ല.
പെ ളികളുറെ സവക രയത:
ഈ പഠനത്തിസല യരഖകൾ െവകോരയത പോലിച്ച് ൊൂക്ഷിക്കും.
വിവരങ്ങൾ
രഹെയമോ ി ൊൂക്ഷിക്കസപ്പെും. ഏസതോരു റിയപ്പോർെിലുസമെിലും
നിങ്ങളുസെ തിരിച്ചറി ൽ ൈോധയമോകുന്ന വിവരങ്ങൾ
ഉൾസപ്പെുത്തുക ില്ല. നിങ്ങളുസെ യപര് അസല്ലെിൽ യഫോൺ നമ്പർ
യചോദിക്കുക ില്ല. ഈ െർയവ ്സക്കോപ്പും യപയരോ തിരിച്ചറി ോ
ോവുന്ന
വിവരങ്ങയളോ ൊൂക്ഷിക്കുക ില്ല. യരഖകൾ യലോക്ക് സചയ്ത ഫ ലിൽ
ൊൂക്ഷിക്കും; ഗ്യവെകരും എതിക്സ ് കമ്മിറ്റി അുംഗ്ങ്ങളുമോണ്
ഇതിയലക്കുള്ള രപയവശനമുള്ളവർ.
ിവെ ർട്ടിംഗും പ്രസിദ്ധീകരണേും:
പഠന ഫലും ഒരു പി ർ റിവയൂ സച ്മത, ഇൻഡക്സഡ് യജർണലിൽ
രപെിദ്ധീകരിക്കുക എന്നതോണ് ഉയേശും. ഫലങ്ങൾ രഗ്ൂപ്പ്
അെിസ്ഥോനത്തിലോണ് കോണിക്കുക, വയക്തിഗ്ത ഡോറ്റ
ഉപയ ോഗ്ിക്കുക ില്ല. പഠനത്തിന്സറ റിയപ്പോർെ് എതിക്സ ് കമ്മിറ്റിക്ക്
െമർപ്പിക്കും.
കൂെുതൽ േിശദീകരണങ്ങൾക്ക് ബന്ധറെെ േുന്നേർ:
വ . റഷയ്ഖ് സ ഫ ത്ത് മുചമ്മിൽ (1-ോും വർെ എും.ഡി.എെ്
വിദയോർത്ഥി)
യഫോൺ: 9381066758
ഇസമ ിൽ: saaffushaik07@gmail.com

വ . ജുനനദ്
റീഡർ
സപോതു ആയരോഗ്യ ദന്തവിദയ വകുപ്പ്
Awareness and attitudes toward risks of “do-it-yourself”
Dental practices: a questionnaire study
Version 3 26-12-2025
Page 9 of 9

സ നയപോ ദന്ത യകോയളജ്, യദരലക്കസെ, മുംഗ്ളൂരു
യഫോൺ: 9964012940
ഇസമ ിൽ: dr.junaid.bantwal@gmail.com
പെ ളിയുറെ അേക ശങ്ങറളക്കുിച്ചുള്ള സംശയങ്ങൾക്ക്
പ്പധ ന ഗവേഷകന്റെ മുെപി തൃപ്ഥികരമറൈിൽ
ബന്ധറൈ േുന്ന എതിക്സ ് കമ്മിറ്റി അംഗത്തിന്റെ
േിശദ ംശങ്ങൾ:
എതിക്സ ് കമ്മിറ്റി:
വ . ലീന പ്പവമ ദ്
സമമ്പർ സെരകെറി
സ നയപോ എതിക്സ ് കമ്മിറ്റി – 2 -2േ-ാമസത്ത സ െ്മസമന്റ്,
സ നയപോ ദന്ത യകോയളജ്
ഇസമ ിൽ: yec2@yenepoya.edu.in
യഫോൺ: 0824-2206000

Language

1. Language *

Mark only one oval.

- ( ) ಕನ್ನಡ *Skip to question 31*
- ( ) മലയാളം *Skip to question 60*
- ( ) English *Skip to question 2*

AWARENESS AND ATTITUDES TOWARD RISKS OF “DO-IT-YOURSELF” DENTAL PRACTICES: A QUESTIONNAIRE STUDY

A **do-it-yourself (DIY) dental procedure** refers to **any dental treatment or oral care practice performed by an individual at home, without direct supervision or intervention by a qualified dental professional**. These procedures are usually guided by **online instructions, social media content, product manuals, or advice from non-professionals**, rather than a dentist.

2. Age *

3. Gender *

Mark only one oval.

- Male
- Female
- Others

4. Education *

Mark only one oval.

- No formal education
- High School
- Intermediate or PUC
- Graduate
- Post graduate

5. Have you ever used any “do-it-yourself” (DIY) dental product or method? *

Mark only one oval.

- Yes
- No

6. If yes, please mention the type used (e.g., whitening kits, aligners, home remedies, etc.): *

7. **I am aware of what “do-it-yourself” dental practices mean.** *

Mark only one oval.

- Strongly disagree
- Disagree
- Neutral
- Agree
- Strongly Agree

8. **I feel confident using home remedies for dental problems.** *

Mark only one oval.

- Strongly disagree
- Disagree
- Neutral
- Agree
- Strongly agree

9. **I believe DIY dental treatments are convenient.** *

Mark only one oval.

- Strongly disagree
- Disagree
- Neutral
- Agree
- Strongly agree

10. **DIY dental options are more affordable than professional dental care.** *

Mark only one oval.

- Strongly disagree
- Disagree
- Neutral
- Agree
- Strongly agree

11. **It is acceptable to try DIY solutions when dental clinics are not easily accessible.** *

Mark only one oval.

- Strongly disagree
- Disagree
- Neutral
- Agree
- Strongly Agree

12. **I am open to using DIY methods if I cannot afford professional dental care.** *

Mark only one oval.

- Strongly disagree
- Disagree
- Neutral
- Agree
- Strongly Agree

13. **DIY dental treatments can lead to complications such as tooth damage or gum injury.** *

Mark only one oval.

- Strongly disagree
- Disagree
- Neutral
- Agree
- Strongly agree

14. **At-home dental kits are not always approved by dental professionals.** *

Mark only one oval.

- Strongly disagree
- Disagree
- Neutral
- Agree
- Strongly agree

15. **Using mail-order dental aligners without professional supervision can be harmful.** *

Mark only one oval.

- Strongly disagree
- Disagree
- Neutral
- Agree
- Strongly agree

16. **Improper use of whitening agents can cause enamel erosion.** *

Mark only one oval.

- Strongly disagree
- Disagree
- Neutral
- Agree
- Strongly agree

17. **There is a lack of professional guidance in most DIY dental methods.** *

Mark only one oval.

- Strongly disagree
- Disagree
- Neutral
- Agree
- Strongly agree

18. **Self-treatment can result in infections or incorrect diagnosis.** *

Mark only one oval.

- Strongly disagree
- Disagree
- Neutral
- Agree
- Strongly Agree

19. **Most people do not fully understand the risks before trying DIY dental solutions.** *

Mark only one oval.

- Strongly disagree
- Disagree
- Neutral
- Agree
- Strongly agree

20. **I have heard of people experiencing side effects after using DIY dental treatments.** *

Mark only one oval.

- Strongly disagree
- Disagree
- Neutral
- Agree
- Strongly agree

21. **Social media platforms often promote unsafe DIY dental hacks.** *

Mark only one oval.

- Strongly disagree
- Disagree
- Neutral
- Agree
- Strongly agree

22. **I trust dental products recommended by social media influencers.** *

Mark only one oval.

- Strongly disagree
- Disagree
- Neutral
- Agree
- Strongly agree

23. **I prefer professional dental consultation rather than treating myself.** *

Mark only one oval.

- Strongly disagree
- Disagree
- Neutral
- Agree
- Strongly agree

24. **DIY dental methods should be avoided unless recommended by dental professionals.** *

Mark only one oval.

- Strongly disagree
- Disagree
- Neutral
- Agree
- Strongly agree

25. **Professional dental care is worth the cost for safety and effective results.** *

Mark only one oval.

- Strongly disagree
- Disagree
- Neutral
- Agree
- Strongly agree

26. **DIY dental treatments should be better regulated.** *

Mark only one oval.

- Strongly Disagree
- Disagree
- Neutral
- Agree
- Strongly agree

27. **There should be more awareness campaigns about the risks of DIY dental care.** *

Mark only one oval.

- Strongly disagree
- Disagree
- Neutral
- Agree
- Strongly agree

28. **DIY treatments are safe if instructions are followed carefully.** *

Mark only one oval.

- Strongly disagree
- Disagree
- Neutral
- Agree
- Strongly agree

29. **I am likely to discourage others from using unsafe DIY dental techniques.** *

Mark only one oval.

- Strongly disagree
- Disagree
- Neutral
- Agree
- Strongly agree

30. **Educational content on the risks of DIY dental practices should be widely promoted.** *

Mark only one oval.

- Strongly disagree
- Disagree
- Neutral
- Agree
- Strongly agree

"ಸ್ವಯಂ ದಂತ ಚಿಕಿತ್ಸೆಗಳ' ಅಪಾಯಗಳ ಬಗ್ಗೆ ಅರಿವು ಮತ್ತು ವರ್ತನೆಗಳು: ಪ್ರಶ್ನಾವಳಿ ಅಧ್ಯಯನ

ಸ್ವಯಂ ದಂತ ಚಿಕಿತ್ಸೆಗಳ (DIY) ಎಂದರೆ ಅರ್ಹ ದಂತ ವೃತ್ತಿಪರರ ನೇರ ಮೇಲ್ವಿಚಾರಣೆ ಅಥವಾ ಹಸ್ತಕ್ಷೇಪವಿಲ್ಲದೆ, ಮನೆಯಲ್ಲಿಯೇ ಒಬ್ಬ ವ್ಯಕ್ತಿಯು ನಡೆಸುವ ಯಾವುದೇ ದಂತ ಚಿಕಿತ್ಸೆ ಅಥವಾ ಮೌಖಿಕ ಆರೈಕೆ ಅಭ್ಯಾಸ. ಈ ಕಾರ್ಯವಿಧಾನಗಳನ್ನು ಸಾಮಾನ್ಯವಾಗಿ ದಂತ ವೈದ್ಯರ ಬದಲಿಗೆ ಆನ್‌ಲೈನ್ ಸೂಚನೆಗಳು, ಸಾಮಾಜಿಕ ಮಾಧ್ಯಮ ವಿಷಯ, ಉತ್ಪನ್ನ ಕೈಪಿಡಿಗಳು ಅಥವಾ ವೃತ್ತಿಪರರಲ್ಲದವರ ಸಲಹೆಯಿಂದ ನಿರ್ದೇಶಿಸಲಾಗುತ್ತದೆ.

31. **ವಯಸ್ಸು** *

32. **ಲಿಂಗ** *

Mark only one oval.

- ಪುರುಷ
- ಮಹಿಳೆ
- ಇತರೆ

33. **ಶಿಕ್ಷಣ ಮಟ್ಟ** *

Mark only one oval.

- ಯಾವುದೇ ಶಿಷ್ಟ ಶಿಕ್ಷಣವಿಲ್ಲ
- ಪ್ರಾಥಮಿಕ
- ಪ್ರೌಢ
- ಪದವಿ
- ಸ್ನಾತಕೋತ್ತರ

34. **ನೀವು ಯಾವುದೇ ' ಸ್ವಯಂ ದಂತ ಚಿಕಿತ್ಸೆಗಳ' ದಂತ ಉತ್ಪನ್ನ ಅಥವಾ ವಿಧಾನ ಬಳಸಿದ್ದೀರಾ?** *

Mark only one oval.

- ಹೌದು
- ಇಲ್ಲ

35. **ಹೌದಾದರೆ, ನೀವು ಬಳಸಿದ ಉತ್ಪನ್ನದ ವಿಧವನ್ನು ನಮೂದಿಸಿ (ಉದಾ: ಹಿಗ್ಗು ಕಿಟ್, ಅಲೈನರ್ ಇತ್ಯಾದಿ):** *

____

36. **" ಸ್ವಯಂ ದಂತ ಚಿಕಿತ್ಸೆಗಳ " ಎಂಬ ದಂತ ಚಿಕಿತ್ಸಾಲಯಗಳ ಅರ್ಥವೇನೆಂದು ನನಗೆ ತಿಳಿದಿದೆ.** *

Mark only one oval.

- ಸಂಪೂರ್ಣವಾಗಿ ಅಸಮ್ಮತ
- ಅಸಮ್ಮತ
- ತಟಸ್ಥ
- ಸಮ್ಮತ
- ಸಂಪೂರ್ಣವಾಗಿ ಸಮ್ಮತ

37. **ನಾನು ದಂತ ಸಮಸ್ಯೆಗಳಿಗೆ ಮನೆಮದ್ದುಗಳನ್ನು ಬಳಸುವಲ್ಲಿ ಆತ್ಮವಿಶ್ವಾಸ ಹೊಂದಿದ್ದೇನೆ.** *

Mark only one oval.

- ಸಂಪೂರ್ಣವಾಗಿ ಅಸಮ್ಮತ
- ಅಸಮ್ಮತ
- ತಟಸ್ಥ
- ಸಮ್ಮತ
- ಸಂಪೂರ್ಣವಾಗಿ ಸಮ್ಮತ

38. **ಸ್ವಯಂ (DIY) ದಂತ ಚಿಕಿತ್ಸೆಗಳು ಅನುಕೂಲಕರವಾಗಿವೆ ಎಂದು ನಾನು ನಂಬುತ್ತೇನೆ.** *

Mark only one oval.

- ಸಂಪೂರ್ಣವಾಗಿ ಅಸಮ್ಮತ
- ಅಸಮ್ಮತ
- ತಟಸ್ಥ
- ಸಮ್ಮತ
- ಸಂಪೂರ್ಣವಾಗಿ ಸಮ್ಮತ

39. **ಸ್ವಯಂ ದಂತ ಚಿಕಿತ್ಸೆಗಳು ವೃತ್ತಿಪರ ದಂತ ಚಿಕಿತ್ಸೆಯಿಗಿಂತ ಕಡಿಮೆ ವೆಚ್ಚವಾಗಿವೆ.** *

Mark only one oval.

- ಸಂಪೂರ್ಣವಾಗಿ ಅಸಮ್ಮತ
- ಅಸಮ್ಮತ
- ತಟಸ್ಥ
- ಸಮ್ಮತ
- ಸಂಪೂರ್ಣವಾಗಿ ಸಮ್ಮತ

40. **ದಂತ ಚಿಕಿತ್ಸಾ ಕೇಂದ್ರಗಳು ಸುಲಭವಾಗಿ ಲಭ್ಯವಿಲ್ಲದಾಗ ಸ್ವಯಂ ಚಿಕಿತ್ಸೆಯನ್ನು ಪ್ರಯತ್ನಿಸುವುದು ಸರಿ ಎಂದು ನಾನು ಭಾವಿಸುತ್ತೇನೆ.** *

Mark only one oval.

- ಸಂಪೂರ್ಣವಾಗಿ ಅಸಮ್ಮತ
- ಅಸಮ್ಮತ
- ತಟಸ್ಥ
- ಸಮ್ಮತ
- ಸಂಪೂರ್ಣವಾಗಿ ಸಮ್ಮತ

41. **ನಾನು ದಂತ ವೈದ್ಯರನ್ನು ಸಂಪರ್ಕಿಸಲು ಹಣದ ಕೊರತೆ ಇದ್ದರೆ ಸ್ವಯಂ ದಂತ ಚಿಕಿತ್ಸೆಯನ್ನು ಬಳಸಲು ಸಿದ್ಧನಾಗಿದ್ದೇನೆ.** *

Mark only one oval.

- ಸಂಪೂರ್ಣವಾಗಿ ಅಸಮ್ಮತ
- ಅಸಮ್ಮತ
- ತಟಸ್ಥ
- ಸಮ್ಮತ
- ಸಂಪೂರ್ಣವಾಗಿ ಸಮ್ಮತ

42. **ಸ್ವಯಂ ದಂತ ಚಿಕಿತ್ಸೆಗಳು ಹಲ್ಲು ಹಾನಿ ಅಥವಾ ಹಲ್ಲುಮೂಳೆ ಗಾಯಗಳಂತಹ ತೊಂದರೆಗಳಿಗೆ ಕಾರಣವಾಗಬಹುದು.** *

Mark only one oval.

- ಸಂಪೂರ್ಣವಾಗಿ ಅಸಮ್ಮತ
- ಅಸಮ್ಮತ
- ತಟಸ್ಥ
- ಸಮ್ಮತ
- ಸಂಪೂರ್ಣವಾಗಿ ಸಮ್ಮತ

43. **ಮನೆಯಲ್ಲೇ ಬಳಸುವ ದಂತ ಕಿಟ್‌ಗಳಿಗೆ ದಂತ ವೈದ್ಯರ ಅನುಮೋದನೆ ಯಾವಾಗಲೂ ಇರುವುದಿಲ್ಲ.** *

Mark only one oval.

- ಸಂಪೂರ್ಣವಾಗಿ ಅಸಮ್ಮತ
- ಅಸಮ್ಮತ
- ತಟಸ್ಥ
- ಸಮ್ಮತ
- ಸಂಪೂರ್ಣವಾಗಿ ಸಮ್ಮತ

44. **ವೃತ್ತಿಪರ ಮೇಲ್ವಿಚಾರಣೆ ಇಲ್ಲದೆ ಮೇಲ್-ಆರ್ಡರ್ ದಂತ ಅಲೈನರ್‌ಗಳನ್ನು ಬಳಸುವುದು ಹಾನಿಕಾರಕವಾಗಬಹುದು.** *

Mark only one oval.

- ಸಂಪೂರ್ಣವಾಗಿ ಅಸಮ್ಮತ
- ಅಸಮ್ಮತ
- ತಟಸ್ಥ
- ಸಮ್ಮತ
- ಸಂಪೂರ್ಣವಾಗಿ ಸಮ್ಮತ

45. **ತಪ್ಪಾಗಿ ಬಳಸಿದ ದಂತ ಬಿಳಿಮಾಡುವ ದ್ರವ್ಯಗಳು ಹಲ್ಲಿನ ಎನಾಮೆಲ್ ಅನ್ನು ಹಾನಿಗೊಳಿಸಬಹುದು.** *

Mark only one oval.

- ಸಂಪೂರ್ಣವಾಗಿ ಅಸಮ್ಮತ
- ಅಸಮ್ಮತ
- ತಟಸ್ಥ
- ಸಮ್ಮತ
- ಸಂಪೂರ್ಣವಾಗಿ ಸಮ್ಮತ

46. **ಬಹುತೇಕ ಸ್ವಯಂ ದಂತ ವಿಧಾನಗಳಲ್ಲಿ ವೃತ್ತಿಪರ ಮಾರ್ಗದರ್ಶನದ ಕೊರತೆ ಇದೆ.** *

Mark only one oval.

- ಸಂಪೂರ್ಣವಾಗಿ ಅಸಮ್ಮತ
- ಅಸಮ್ಮತ
- ತಟಸ್ಥ
- ಸಮ್ಮತ
- ಸಂಪೂರ್ಣವಾಗಿ ಸಮ್ಮತ

47. **ಸ್ವಯಂ ಚಿಕಿತ್ಸೆ ಸೋಂಕುಗಳು ಅಥವಾ ತಪ್ಪು ನಿರ್ಧಾರಗಳಿಗೆ ಕಾರಣವಾಗಬಹುದು.** *

Mark only one oval.

- ಸಂಪೂರ್ಣವಾಗಿ ಅಸಮ್ಮತ
- ಅಸಮ್ಮತ
- ತಟಸ್ಥ
- ಸಮ್ಮತ
- ಸಂಪೂರ್ಣವಾಗಿ ಸಮ್ಮತ

48. **ಸ್ವಯಂ ದಂತ ಚಿಕಿತ್ಸೆಯನ್ನು ಪ್ರಯತ್ನಿಸುವ ಮೊದಲು ಹೆಚ್ಚಿನ ಜನರಿಗೆ ಅದರ ಅಪಾಯಗಳ ಬಗ್ಗೆ ಪೂರ್ಣ ಅರಿವು ಇರುವುದಿಲ್ಲ.** *

Mark only one oval.

- ಸಂಪೂರ್ಣವಾಗಿ ಅಸಮ್ಮತ
- ಅಸಮ್ಮತ
- ತಟಸ್ಥ
- ಸಮ್ಮತ
- ಸಂಪೂರ್ಣವಾಗಿ ಸಮ್ಮತ

49. **ಸ್ವಯಂ ದಂತ ಚಿಕಿತ್ಸೆಗಳ ಬಳಕೆಯಿಂದ ಕೆಲವರು ಅಡ್ಡ ಪರಿಣಾಮಗಳನ್ನು ಅನುಭವಿಸಿರುವ ಬಗ್ಗೆ ನಾನು ಕೇಳಿದ್ದೇನೆ.** *

Mark only one oval.

- ಸಂಪೂರ್ಣವಾಗಿ ಅಸಮ್ಮತ
- ಅಸಮ್ಮತ
- ತಟಸ್ಥ
- ಸಮ್ಮತ
- ಸಂಪೂರ್ಣವಾಗಿ ಸಮ್ಮತ

50. **ಸಾಮಾಜಿಕ ಮಾಧ್ಯಮ ವೇದಿಕೆಗಳು ಅಪಾಯಕಾರಿ ಸ್ವಯಂ ದಂತ ವಿಧಾನಗಳನ್ನು ಪ್ರಚಾರ ಮಾಡುತ್ತವೆ.** *

Mark only one oval.

- ಸಂಪೂರ್ಣವಾಗಿ ಅಸಮ್ಮತ
- ಅಸಮ್ಮತ
- ತಟಸ್ಥ
- ಸಮ್ಮತ
- ಸಂಪೂರ್ಣವಾಗಿ ಸಮ್ಮತ

51. **ಸಾಮಾಜಿಕ ಮಾಧ್ಯಮದ ಪ್ರಭಾವಿಗಳಿಂದ ಶಿಫಾರಸು ಮಾಡಲಾದ ದಂತ ಉತ್ಪನ್ನಗಳನ್ನು ನಾನು ನಂಬುತ್ತೇನೆ.** *

Mark only one oval.

- ಸಂಪೂರ್ಣವಾಗಿ ಅಸಮ್ಮತ
- ಅಸಮ್ಮತ
- ತಟಸ್ಥ
- ಸಮ್ಮತ
- ಸಂಪೂರ್ಣವಾಗಿ ಸಮ್ಮತ

52. **ನಾನು ಸ್ವಯಂ ಚಿಕಿತ್ಸೆಗಿಂತ ವೃತ್ತಿಪರ ದಂತ ಸಲಹೆಯನ್ನು ಆದ್ಯತೆಯಾಗಿ ಆಯ್ಕೆಮಾಡುತ್ತೇನೆ.** *

Mark only one oval.

- ಸಂಪೂರ್ಣವಾಗಿ ಅಸಮ್ಮತ
- ಅಸಮ್ಮತ
- ತಟಸ್ಥ
- ಸಮ್ಮತ
- ಸಂಪೂರ್ಣವಾಗಿ ಸಮ್ಮತ

53. **ದಂತ ವೈದ್ಯರು ಶಿಫಾರಸು ಮಾಡದೇ ಇದ್ದರೆ ಸ್ವಯಂ ದಂತ ವಿಧಾನಗಳನ್ನು ತಪ್ಪಿಸಬೇಕು.** *

Mark only one oval.

- ಸಂಪೂರ್ಣವಾಗಿ ಅಸಮ್ಮತ
- ಅಸಮ್ಮತ
- ತಟಸ್ಥ
- ಸಮ್ಮತ
- ಸಂಪೂರ್ಣವಾಗಿ ಸಮ್ಮತ

54. **ಸುರಕ್ಷತೆ ಮತ್ತು ಪರಿಣಾಮಕಾರಿ ಫಲಿತಾಂಶಗಳಿಗಾಗಿ ವೃತ್ತಿಪರ ದಂತ ಚಿಕಿತ್ಸೆ ವೆಚ್ಚಕ್ಕೆ ತಕ್ಕದ್ದೇ ಆಗಿದೆ.** *

Mark only one oval.

- ಸಂಪೂರ್ಣವಾಗಿ ಅಸಮ್ಮತ
- ಅಸಮ್ಮತ
- ತಟಸ್ಥ
- ಸಮ್ಮತ
- ಸಂಪೂರ್ಣವಾಗಿ ಸಮ್ಮತ

55. **ಸ್ವಯಂ ದಂತ ಚಿಕಿತ್ಸೆಗಳು ಉತ್ತಮವಾಗಿ ನಿಯಂತ್ರಿಸಲ್ಪಡಬೇಕು ಎಂದು ನಾನು ನಂಬುತ್ತೇನೆ.** *

Mark only one oval.

- ಸಂಪೂರ್ಣವಾಗಿ ಅಸಮ್ಮತ
- ಅಸಮ್ಮತ
- ತಟಸ್ಥ
- ಸಮ್ಮತ
- ಸಂಪೂರ್ಣವಾಗಿ ಸಮ್ಮತ

56. **ಸ್ವಯಂ ದಂತ ಚಿಕಿತ್ಸೆಗಳ ಅಪಾಯಗಳ ಬಗ್ಗೆ ಹೆಚ್ಚಿನ ಜಾಗೃತಿ ಅಭಿಯಾನಗಳು ಅಗತ್ಯವಿವೆ.** *

Mark only one oval.

- ಸಂಪೂರ್ಣವಾಗಿ ಅಸಮ್ಮತ
- ಅಸಮ್ಮತ
- ತಟಸ್ಥ
- ಸಮ್ಮತ
- ಸಂಪೂರ್ಣವಾಗಿ ಸಮ್ಮತ

57. **ಸೂಚನೆಗಳನ್ನು ಸರಿಯಾಗಿ ಪಾಲಿಸಿದರೆ ಸ್ವಯಂ ದಂತ ಚಿಕಿತ್ಸೆಗಳು ಸುರಕ್ಷಿತವಾಗಿವೆ ಎಂದು ನಾನು ಭಾವಿಸುತ್ತೇನೆ.** *

Mark only one oval.

- ಸಂಪೂರ್ಣವಾಗಿ ಅಸಮ್ಮತ
- ಅಸಮ್ಮತ
- ತಟಸ್ಥ
- ಸಮ್ಮತ
- ಸಂಪೂರ್ಣವಾಗಿ ಸಮ್ಮತ

58. **ಅಪಾಯಕಾರಿ ಸ್ವಯಂ ದಂತ ವಿಧಾನಗಳನ್ನು ಬಳಸುವುದನ್ನು ಇತರರಿಗೆ ನಾನು ತಡೆಯುವ ಸಾಧ್ಯತೆ ಇದೆ.** *

Mark only one oval.

- ಸಂಪೂರ್ಣವಾಗಿ ಅಸಮ್ಮತ
- ಅಸಮ್ಮತ
- ತಟಸ್ಥ
- ಸಮ್ಮತ
- ಸಂಪೂರ್ಣವಾಗಿ ಸಮ್ಮತ

59. **ಸ್ವಯಂ ದಂತ ಚಿಕಿತ್ಸೆಗಳ ಅಪಾಯಗಳ ಬಗ್ಗೆ ಶಿಕ್ಷಣಾತ್ಮಕ ಮಾಹಿತಿಯನ್ನು ವ್ಯಾಪಕವಾಗಿ ಪ್ರಚಾರ ಮಾಡಬೇಕು ಎಂದು ನಾನು ಭಾವಿಸುತ್ತೇನೆ.** *

Mark only one oval.

- ಸಂಪೂರ್ಣವಾಗಿ ಅಸಮ್ಮತ
- ಅಸಮ್ಮತ
- ತಟಸ್ಥ
- ಸಮ್ಮತ
- ಸಂಪೂರ್ಣವಾಗಿ ಸಮ್ಮತ

"സ്വയം ചെയ്യുക" എന്ന ദന്തചികിത്സയുടെ അപകടസാധ്യതകളെക്കുറിച്ചുള്ള അവബോധവും മനോഭാവങ്ങളും: ഒരു ചോദ്യാവലി പഠനം

ഒരു ഡു-ഇറ്റ്-സ്വയം (DIY) ഡെന്റൽ നടപടിക്രമം എന്നത് ഒരു യോഗ്യതയുള്ള ദന്ത വിദഗ്ദ്ധന്റെ നേരിട്ടുള്ള മേൽനോട്ടമോ ഇടപെടലോ ഇല്ലാതെ വീട്ടിൽ ഒരു വ്യക്തി നടത്തുന്ന ഏതെങ്കിലും ദന്ത ചികിത്സയെയോ ഓറൽ കെയർ പ്രാക്ടീസിനെയോ സൂചിപ്പിക്കുന്നു. ഈ നടപടിക്രമങ്ങൾ സാധാരണയായി ഒരു ദന്തരോഗവിദഗ്ദ്ധന് പകരം ഓൺലൈൻ നിർദ്ദേശങ്ങൾ, സോഷ്യൽ മീഡിയ ഉള്ളടക്കം, ഉൽപ്പന്ന മാനുവലുകൾ അല്ലെങ്കിൽ പ്രൊഫഷണലുകൾ അല്ലാത്തവരുടെ ഉപദേശം എന്നിവയെ അടിസ്ഥാനമാക്കിയാണ് നടത്തുന്നത്.

60. **പ്രായം** *

61. **ലിംഗം** *

Mark only one oval.

- പുരുഷൻ
- സ്ത്രീ
- മറ്റ്

62. **വിദ്യാഭ്യാസ നിലവാരം** *

Mark only one oval.

- ഔപചാരിക വിദ്യാഭ്യാസമില്ല
- പ്രാഥമിക
- സെക്കൻഡറി
- ബിരുദം
- ബിരുദാനന്തര

63. **നിങ്ങൾ ഒരിക്കലെങ്കിലും “സ്വയം ചെയ്യുക” (DIY) ദന്ത ഉൽപ്പന്നമോ ചികിത്സാ രീതിയോ ഉപയോഗിച്ചിട്ടുണ്ടോ?** *

Mark only one oval.

- അതെ
- ഇല്ല

64. **അതെ എങ്കിൽ, ഉപയോഗിച്ച തരങ്ങൾ സൂചിപ്പിക്കുക (ഉദാ: പല്ല് വെളുപ്പിക്കുന്ന കിറ്റുകൾ, അലൈണറുകൾ, വീട്ടുവൈദ്യങ്ങൾ മുതലായവ)** *

65. **“സ്വയം ചെയ്യുക” (DIY) ദന്ത ചികിത്സകൾ എന്താണെന്ന് എനിക്ക് അറിയാം.** *

Mark only one oval.

- ശക്തമായി അസമ്മതിക്കുന്നു
- അസമ്മതിക്കുന്നു
- നിഷ്പക്ഷം
- സമ്മതിക്കുന്നു
- ശക്തമായി സമ്മതിക്കുന്നു

66. **ദന്ത പ്രശ്നങ്ങൾക്ക് വീട്ടുവൈദ്യങ്ങൾ ഉപയോഗിക്കാൻ എനിക്ക് ആത്മവിശ്വാസമുണ്ട്.** *

Mark only one oval.

- ശക്തമായി അസമ്മതിക്കുന്നു
- അസമ്മതിക്കുന്നു
- നിഷ്പക്ഷം
- സമ്മതിക്കുന്നു
- ശക്തമായി സമ്മതിക്കുന്നു

67. **സ്വയം (DIY) ദന്ത ചികിത്സകൾ സൗകര്യപ്രദമാണെന്ന് ഞാൻ വിശ്വസിക്കുന്നു.** *

Mark only one oval.

- ശക്തമായി അസമ്മതിക്കുന്നു
- അസമ്മതിക്കുന്നു
- നിഷ്പക്ഷം
- സമ്മതിക്കുന്നു
- ശക്തമായി സമ്മതിക്കുന്നു

68. **സ്വയം ദന്ത ചികിത്സകൾ പ്രൊഫഷണൽ ദന്ത ചികിത്സയേക്കാൾ ചെലവുകുറവാണ്.** *

Mark only one oval.

- ശക്തമായി അസമ്മതിക്കുന്നു
- അസമ്മതിക്കുന്നു
- നിഷ്പക്ഷം
- സമ്മതിക്കുന്നു
- ശക്തമായി സമ്മതിക്കുന്നു

69. **ദന്ത ചികിത്സാ കേന്ദ്രങ്ങൾ എളുപ്പത്തിൽ ലഭ്യമല്ലാത്തപ്പോൾ സ്വയം ചികിത്സകൾ പരീക്ഷിക്കുന്നത് അംഗീകരിക്കാവുന്നതാണ്.** *

Mark only one oval.

- ശക്തമായി അസമ്മതിക്കുന്നു
- അസമ്മതിക്കുന്നു
- നിഷ്പക്ഷം
- സമ്മതിക്കുന്നു
- ശക്തമായി സമ്മതിക്കുന്നു

70. **ദന്ത ചികിത്സയ്ക്ക് സാമ്പത്തികമായി കഴിയാത്ത സാഹചര്യത്തിൽ സ്വയം ദന്ത ചികിത്സകൾ ഉപയോഗിക്കാൻ ഞാൻ തയ്യാറാണ്.** *

Mark only one oval.

- ശക്തമായി അസമ്മതിക്കുന്നു
- അസമ്മതിക്കുന്നു
- നിഷ്പക്ഷം
- സമ്മതിക്കുന്നു
- ശക്തമായി സമ്മതിക്കുന്നു

71. **സ്വയം ദന്ത ചികിത്സകൾ പല്ല് കേടുപാടുകൾക്കും മസൂകുകൾക്ക് പരിക്കുകൾക്കും കാരണമാകാം.** *

Mark only one oval.

- ശക്തമായി അസമ്മതിക്കുന്നു
- അസമ്മതിക്കുന്നു
- നിഷ്പക്ഷം
- സമ്മതിക്കുന്നു
- ശക്തമായി സമ്മതിക്കുന്നു

72. **വീട്ടിൽ ഉപയോഗിക്കുന്ന ദന്ത കിറ്റുകൾക്ക് എല്ലായ്പ്പോഴും ദന്തഡോക്ടർമാരുടെ അംഗീകാരം ഉണ്ടായിരിക്കണമെന്നില്ല.** *

Mark only one oval.

- ശക്തമായി അസമ്മതിക്കുന്നു
- അസമ്മതിക്കുന്നു
- നിഷ്പക്ഷം
- സമ്മതിക്കുന്നു
- ശക്തമായി സമ്മതിക്കുന്നു

73. **പ്രൊഫഷണൽ മേൽനോട്ടമില്ലാതെ മെയിൽ-ഓർഡർ ദന്ത അലൈണറുകൾ ഉപയോഗിക്കുന്നത് ഹാനികരമായിരിക്കാം.** *

Mark only one oval.

- ശക്തമായി അസമ്മതിക്കുന്നു
- അസമ്മതിക്കുന്നു
- നിഷ്പക്ഷം
- സമ്മതിക്കുന്നു
- ശക്തമായി സമ്മതിക്കുന്നു

74. **തെറ്റായി ഉപയോഗിക്കുന്ന പല്ല് വെളുപ്പിക്കുന്ന വസ്തുക്കൾ പല്ലിന്റെ എൻാമലിനെ നശിപ്പിക്കാം.** *

Mark only one oval.

- ശക്തമായി അസമ്മതിക്കുന്നു
- അസമ്മതിക്കുന്നു
- നിഷ്പക്ഷം
- സമ്മതിക്കുന്നു
- ശക്തമായി സമ്മതിക്കുന്നു

75. **മിക്ക സ്വയം ദന്ത ചികിത്സാ രീതികളിലും പ്രൊഫഷണൽ മാർഗ്ഗനിർദേശത്തിന്റെ കുറവുണ്ട്.** *

Mark only one oval.

- ശക്തമായി അസമ്മതിക്കുന്നു
- അസമ്മതിക്കുന്നു
- നിഷ്പക്ഷം
- സമ്മതിക്കുന്നു
- ശക്തമായി സമ്മതിക്കുന്നു

76. **സ്വയം ചികിത്സ അണുബാധകളിലേക്കോ തെറ്റായ രോഗനിർണയത്തിലേക്കോ നയിക്കാം.** *

Mark only one oval.

- ശക്തമായി അസമ്മതിക്കുന്നു
- അസമ്മതിക്കുന്നു
- നിഷ്പക്ഷം
- സമ്മതിക്കുന്നു
- ശക്തമായി സമ്മതിക്കുന്നു

77. **സ്വയം ദന്ത ചികിത്സകൾ പരീക്ഷിക്കുന്നതിന് മുമ്പ് മിക്ക ആളുകൾക്കും അതിന്റെ അപകടങ്ങളെക്കുറിച്ച് പൂർണ്ണമായ അറിവില്ല.** *

Mark only one oval.

- ശക്തമായി അസമ്മതിക്കുന്നു
- അസമ്മതിക്കുന്നു
- നിഷ്പക്ഷം
- സമ്മതിക്കുന്നു
- ശക്തമായി സമ്മതിക്കുന്നു

78. **സ്വയം ദന്ത ചികിത്സകൾ ഉപയോഗിച്ചതിന് ശേഷം ചിലർ പാർശ്വഫലങ്ങൾ അനുഭവിച്ചിട്ടുണ്ടെന്ന് ഞാൻ കേട്ടിട്ടുണ്ട്.** *

Mark only one oval.

- ശക്തമായി അസമ്മതിക്കുന്നു
- അസമ്മതിക്കുന്നു
- നിഷ്പക്ഷം
- സമ്മതിക്കുന്നു
- ശക്തമായി സമ്മതിക്കുന്നു

79. **സാമൂഹ്യ മാധ്യമ പ്ലാറ്റ്ഫോമുകൾ അപകടകരമായ സ്വയം ദന്ത ചികിത്സാ രീതികളെ പ്രചരിപ്പിക്കുന്നു.** *

Mark only one oval.

- ശക്തമായി അസമ്മതിക്കുന്നു
- അസമ്മതിക്കുന്നു
- നിഷ്പക്ഷം
- സമ്മതിക്കുന്നു
- ശക്തമായി സമ്മതിക്കുന്നു

80. **സാമൂഹ്യ മാധ്യമ ഇൻഫ്ലുവൻസർമാർ ശുപാർശ ചെയ്യുന്ന ദന്ത ഉൽപ്പന്നങ്ങളെ ഞാൻ വിശ്വസിക്കുന്നു.** *

Mark only one oval.

- ശക്തമായി അസമ്മതിക്കുന്നു
- അസമ്മതിക്കുന്നു
- നിഷ്പക്ഷം
- സമ്മതിക്കുന്നു
- ശക്തമായി സമ്മതിക്കുന്നു

81. **സ്വയം ചികിത്സയ്ക്കുപകരം പ്രൊഫഷണൽ ദന്തോപദേശം എനിക്ക് മുൻഗണനയാണ്.** *

Mark only one oval.

- ശക്തമായി അസമ്മതിക്കുന്നു
- അസമ്മതിക്കുന്നു
- നിഷ്പക്ഷം
- സമ്മതിക്കുന്നു
- ശക്തമായി സമ്മതിക്കുന്നു

82. **സ്വയം ചികിത്സയ്ക്കുപകരം പ്രൊഫഷണൽ ദന്തോപദേശം എനിക്ക് മുൻഗണനയാണ്.** *

Mark only one oval.

- ശക്തമായി അസമ്മതിക്കുന്നു
- അസമ്മതിക്കുന്നു
- നിഷ്പക്ഷം
- സമ്മതിക്കുന്നു
- ശക്തമായി സമ്മതിക്കുന്നു

83. **ദന്തഡോക്ടർ ശുപാർശ ചെയ്തില്ലെങ്കിൽ സ്വയം ദന്ത ചികിത്സകൾ ഒഴിവാക്കണം.** *

Mark only one oval.

- ശക്തമായി അസമ്മതിക്കുന്നു
- അസമ്മതിക്കുന്നു
- നിഷ്പക്ഷം
- സമ്മതിക്കുന്നു
- ശക്തമായി സമ്മതിക്കുന്നു

84. **സുരക്ഷയും ഫലപ്രദമായ ഫലങ്ങളും ലഭിക്കാൻ പ്രൊഫഷണൽ ദന്ത ചികിത്സ ചെലവിന് വിലമതിക്കുന്നു.** *

Mark only one oval.

- ശക്തമായി അസമ്മതിക്കുന്നു
- അസമ്മതിക്കുന്നു
- നിഷ്പക്ഷം
- സമ്മതിക്കുന്നു
- ശക്തമായി സമ്മതിക്കുന്നു

85. **സ്വയം ദന്ത ചികിത്സകൾ കൂടുതൽ കർശനമായി നിയന്ത്രിക്കപ്പെടണം എന്ന് ഞാൻ വിശ്വസിക്കുന്നു.** *

Mark only one oval.

- ശക്തമായി അസമ്മതിക്കുന്നു
- അസമ്മതിക്കുന്നു
- നിഷ്പക്ഷം
- സമ്മതിക്കുന്നു
- ശക്തമായി സമ്മതിക്കുന്നു

86. **സ്വയം ദന്ത ചികിത്സകളുടെ അപകടങ്ങളെക്കുറിച്ച് കൂടുതൽ ബോധവത്കരണ പ്രചാരണങ്ങൾ ആവശ്യമുണ്ട്.** *

Mark only one oval.

- ശക്തമായി അസമ്മതിക്കുന്നു
- അസമ്മതിക്കുന്നു
- നിഷ്പക്ഷം
- സമ്മതിക്കുന്നു
- ശക്തമായി സമ്മതിക്കുന്നു

87. **നിർദ്ദേശങ്ങൾ കൃത്യമായി പാലിച്ചാൽ സ്വയം ദന്ത ചികിത്സകൾ സുരക്ഷിതമാണെന്ന് ഞാൻ കരുതുന്നു.** *

Mark only one oval.

- ശക്തമായി അസമ്മതിക്കുന്നു
- അസമ്മതിക്കുന്നു
- നിഷ്പക്ഷം
- സമ്മതിക്കുന്നു
- ശക്തമായി സമ്മതിക്കുന്നു

88. **അപകടകരമായ സ്വയം ദന്ത ചികിത്സാ രീതികൾ ഉപയോഗിക്കുന്നത് ഞാൻ മറ്റുള്ളവരെ പിന്തിരിപ്പിക്കാൻ സാധ്യതയുണ്ട്.** *

Mark only one oval.

- ശക്തമായി അസമ്മതിക്കുന്നു
- അസമ്മതിക്കുന്നു
- നിഷ്പക്ഷം
- സമ്മതിക്കുന്നു
- ശക്തമായി സമ്മതിക്കുന്നു

89. **സ്വയം ദന്ത ചികിത്സകളുടെ അപകടങ്ങളെക്കുറിച്ചുള്ള വിദ്യാഭ്യാസ വിവരങ്ങൾ വ്യാപകമായി പ്രചരിപ്പിക്കണം എന്ന് ഞാൻ കരുതുന്നു.** *

Mark only one oval.

- ശക്തമായി അസമ്മതിക്കുന്നു
- അസമ്മതിക്കുന്നു
- നിഷ്പക്ഷം
- സമ്മതിക്കുന്നു
- ശക്തമായി സമ്മതിക്കുന്നു

This content is neither created nor endorsed by Google.

Google Forms
